# Dose-optimized microbial inoculants reshape grape rhizosphere microbiota and enhance fruit quality

**DOI:** 10.3389/fmicb.2025.1702884

**Published:** 2025-11-05

**Authors:** Xiaojian Chang, Ji Chen, Kegang Zhao, Tao Wang, Yong Yang, Xinyue Jia, Bingbing Hu, Yanmei Yu, Fangxiang Li, Yanhui He, Zhansheng Wu

**Affiliations:** ^1^Agricultural Technology Extension Center of Xi'an, Xi'an, China; ^2^Xi'an Key Laboratory of Textile Chemical Engineering Auxiliaries, School of Environmental and Chemical Engineering, Xi'an Polytechnic University, Xi'an, China

**Keywords:** grape, microbial agent, rhizosphere microorganisms, dose-dependent response, soil-plant-fruit

## Abstract

**Introduction:**

Soil serves as a critical habitat for plant growth, harboring diverse microbial communities that profoundly influence soil quality, plant health, and fruit quality. This work aimed to evaluate the potential of microbial inoculants application at varying doses on improving soil health, enhance plant stress resistance, and promote fruit quality.

**Methods:**

Rhizosphere microbial communities across five treatment groups—CK (no inoculant), T1 (45 L/ha), T2 (90 L/ha), T3 (135 L/ha), and T4 (180 L/ha)—were analyzed using high-throughput sequencing on the Illumina NextSeq platform. Concurrently, soil physicochemical properties from the rhizosphere, alongside physiological and biochemical parameters of grape leaves and fruit quality indicators, were measured for all treatments.

**Results:**

The results indicated that inoculant application significantly increased the relative abundance of bacterial phyla Proteobacteria and Actinobacteria, along with the genera *Bacillus, Nitrospira*, and *Pseudomonas*. Concurrently, a decrease in the relative abundance of fungi from the phylum Ascomycota (e.g., *Penicillium, Fusarium*) was observed, whereas an increase was noted for phyla Mortierellomycota and Basidiomycota, and genera such as *Mortierella* and *Solicoccozyma*. Furthermore, microbial inoculant applications (T1–T4) led to significant changes in soil parameters (e.g., nutrient availability and enzyme activity), plant physiological indicators (e.g., antioxidant enzyme activity), and fruit quality metrics (e.g., soluble sugars and vitamin C content). The most pronounced changes were observed under the T2 treatment (90 L/ha), whereas higher doses (T3–T4) resulted in diminished responses together with increased costs, indicating a clear dose-dependent effect.

**Conclusion:**

In summary, the application of compound microbial inoculants, particularly at optimized doses, was associated with shifts in rhizosphere microbial community structure and improvements in soil–plant system metrics, suggesting the potential to contribute to sustainable agricultural management.

## Highlights

- Microbial inoculant application reshaped the rhizosphere microbial community.
- Inoculants enhanced soil quality, plant stress tolerance, and fruit quality.
- Optimal results were achieved with the T2 treatment (90 L/ha).

## 1 Introduction

Grape, a horticultural crop of global importance, is cultivated for its fruit, which serves a wide range of purposes including wine production, fresh consumption, and the extraction of phytochemicals (Strafella et al., 2021). ‘Shine Muscat' has emerged as a highly esteemed table grape cultivar in global markets, renowned for its exceptionally large oval berries, attractive golden-yellow skin, firm yet crisp texture, and rich muscat aroma (Qi et al., 2024). However, despite its commercial success, the sustainable cultivation of ‘Shine Muscat' still faces significant challenges in terms of yield stability and fruit quality, with long-term continuous cropping obstacles being the primary limiting factor. Prolonged monoculture, excessive agrochemical application, and unsustainable farm management have contributed to widespread soil degradation—manifested as compaction, salinization, and nutrient imbalances—as well as the accumulation of autotoxic compounds derived from root exudates and residual plant matter, and the disruption of rhizosphere microbial communities (Chen et al., 2022; Henry et al., 2019; Kayikcioglu et al., 2020; Singh and Kumar, 2021). These alterations not only lead to the proliferation of soil-borne pathogens (such as *Fusarium* and *Verticillium*) but also inhibit the activity of beneficial microorganisms, including mycorrhizal fungi and nitrogen-fixing bacteria. Ultimately, this significantly reduces nutrient uptake efficiency, plant resilience, and overall berry quality (Man et al., 2022; Mazumder et al., 2025).

Microbial inoculants containing plant growth-promoting rhizobacteria (PGPR) represent a promising approach to mitigating these constraints through the improvement of nutrient acquisition, regulation of plant physiological processes, and enhancement of soil ecosystem functionality (He et al., 2024a; Zhang et al., 2024). PGPR strains (such as *Klebsiella oxytoca* Rs-5 and *Bacillus subtilis* SL-44) exert their plant growth-promoting effects through multiple mechanisms: they can directly stimulate plant growth by synthesizing phytohormones (e.g., indole-3-acetic acid) and enhance nutrient acquisition via phosphorus solubilization and biological nitrogen fixation; simultaneously, they inhibit plant pathogens by producing antimicrobial compounds and competing for ecological niches, while inducing systemic resistance in plants (Wu et al., 2019; Yue et al., 2007). Field-based investigations have established the efficacy of plant growth-promoting rhizobacteria (PGPR) in mitigating continuous cropping challenges and improving soil—plant system health in perennial cropping systems (Mohanty et al., 2021). In recent years, field trials have shown that PGPR has a significant effect in alleviating continuous cropping obstacles in perennial crops. For example, studies on *Schisandra chinensis* have shown that microbial inoculants can not only regulate soil microbial communities, but also improve soil fertility and promote plant growth (Li et al., 2024b). Similarly, Ahsan et al. and Wang et al. demonstrated that the application of these microbial inoculants not only enhanced the abundance of beneficial microorganisms but also restructured the rhizosphere microbial community by modifying soil physicochemical properties (including available phosphorus, organic matter, available potassium, and pH, among others) and plant-microbe interactions (Ahsan et al., 2024; Wang et al., 2023b). Furthermore, Zhang et al. showed that *Bacillus velezensis* BY6 enhanced poplar growth while suppressing *Armillaria* pathogens, underscoring its dual role in growth promotion and biocontrol (Zhang et al., 2022).

While microbial inoculants offer a promising solution, their application in contemporary grapevine cultivation are commonly applied at dosages derived from empirical observation rather than rigorous scientific evidence. Simply increasing the application rate does not consistently enhance efficacy and may even induce detrimental effects. A pronounced lack of well-designed field studies persists regarding the influence of inoculant dosage on crop performance, particularly concerning its underlying mechanisms. This knowledge gap substantially limits the development of evidence-based application guidelines. More specifically, the mechanisms through which varying inoculation rates modulate rhizosphere microbial diversity, soil health, plant stress tolerance, and fruit quality remain incompletely understood. Excessive application may disrupt indigenous microbial communities, while insufficient doses often fail to elicit beneficial plant–microbe interactions (Mawarda et al., 2020).

Therefore, this study aimed to systematically investigated the dose-dependent effects of a plant growth-promoting rhizobacterial (PGPR) consortium, comprising *Klebsiella oxytoca* Rs-5 and *Bacillus subtilis* SL-44, on multiple components of the cropping system through field trials conducted in a vineyard system. Specifically, the effects of the inoculant were evaluated across several key aspects: soil properties, including organic matter content, enzymatic activities, and NPK nutrient availability; plant physiological responses, reflected in antioxidant enzyme activity, proline content, and malondialdehyde levels; and berry quality traits, such as individual berry fresh weight, soluble solids content, and sugar-acid ratio. In addition, high-throughput sequencing of 16S rRNA and ITS regions was employed to analyze structural changes within the rhizosphere microbial community. The findings may provide crucial insights into the ecological mechanisms governing PGPR dose effects, thereby establishing a theoretical foundation for developing integrated “bio-soil-plant” management strategies to address continuous cropping challenges, thereby contributing to the achievement of green, efficient, and sustainable viticulture.

## 2 Materials and methods

### 2.1 Materials

The plant growth-promoting rhizobacteria (PGPR) strains *Klebsiella oxytoca* Rs-5 and *Bacillus subtilis* SL-44, previously isolated from the rhizosphere soil of chili plants in Xinjiang saline-alkali soils and preserved in Xi'an Key Laboratory of Textile Chemical Engineering Auxiliaries, exhibited multifunctional traits. Strain Rs-5 demonstrated the ability to produce ACC deaminase, solubilize phosphate, fix nitrogen, and secrete indole-3-acetic acid (IAA) and siderophores (Yue et al., 2007). Strain SL-44 exhibited phosphate solubilization, nitrogen fixation, biofilm formation, and siderophore production (Wu et al., 2019). Strains SL-44 and Rs-5 were individually fermented in LB medium (10 g/L tryptone, 5 g/L yeast extract, 10 g/L NaCl, pH 7.0) in a 50-L bioreactor under controlled conditions: 30 °C, 180 rpm, pH 7.0, and 30% dissolved oxygen for 48 h. All medium components were purchased from Titan Scientific Co., Ltd. (Shanghai, China). The final viable counts reached 5 × 108 CFU/mL for SL-44 and 4 × 10^9^ CFU/mL for Rs-5. Equal volumes of each culture were mixed to form the compound inoculant prior to application.

### 2.2 Field experiment

The field experiment was conducted in Xi‘an Yizhao Modern Agricultural Park (Huyi District, Xi'an, Shaanxi Province, China; 34.05 °N, 108.51 °E), characterized by favorable natural conditions (light, heat, water, air, and soil) and a warm temperate semi-humid continental monsoon climate with distinct seasonal variations. The region has an average annual frost-free period of 216 days, total annual sunshine duration of 1,983.4 hours, mean annual temperature of 13.5 °C, and annual precipitation of 627.6 mm. The experimental field, subjected to long-term continuous monocropping, exhibited localized soil compaction, with baseline soil physicochemical properties detailed in Supplementary Table S1. The experiment was conducted in a 0.4-ha vineyard managed in a single-row system with a planting density of 1500 plants/ha (vine spacing: 1–2 m; row spacing: 4–5 m). Each experimental plot contained 40 consecutive vines, with a 4-meter buffer zone between adjacent plots and one isolation row between different treatments to prevent cross-contamination. A completely randomized design was used, which included five treatments with three replications each (*n* = 15). The treatments included a gradient of compound microbial inoculant concentrations—CK (no inoculant), T1 (45 L/ha), T2 (90 L/ha), T3 (135 L/ha), and T4 (180 L/ha). These inoculant dosages established based on guidelines from preliminary trials and commercial recommendations. The trial began on 5 November 2023. Following local practices, the inoculant was applied four times at critical phenological stages: overwintering (5 November 2023), budburst (28 March 2024), flowering (2 May 2024), and fruit expansion (20 June 2024) based on the nutritional requirements of grape. For each application, a trench (20–30 cm deep) was dug along the furrows, the inoculant was applied, and the trench was backfilled.

At harvest maturity (10 September 2024), composite soil and plant samples were collected from each of the 15 experimental plots (5 treatments × 3 replications). Each composite soil sample consisted of five sub-cores collected from the root zone (20–30 cm depth), with a final weight of approximately 1.0 kg. For plant tissues, five vines per plot were sub-sampled: five leaves and three berry clusters from each vine were pooled to form one composite leaf sample and one composite berry sample per plot, respectively. All samples were immediately placed on ice and transported to the laboratory for processing. Soil samples were sieved (2 mm mesh) to remove stones and plant debris, with one aliquot stored at −80 °C for DNA analysis and another air-dried for chemical properties. Plant samples were rinsed with deionized water, flash-frozen in liquid nitrogen, and ground to a fine powder for subsequent physiological and biochemical analyses. This sampling design provided three biological replicates (*n* = 3) per treatment for all subsequent analyses.

### 2.3 Soil enzyme activity and soil physicochemical properties

Soil enzyme activities were analyzed using fresh samples and expressed on a dry weight basis (mg/g/d) (Zhao et al., 2018). For each enzyme assay, three biological replicates were performed. Alkaline phosphatase (ALP) activity was determined by incubating 1 g of soil with 0.5 mL of 0.05 M p-nitrophenyl phosphate (Titan Scientific Co., Ltd., Shanghai, China) in 4 mL of universal buffer (pH 11.0) at 37 °C for 1 h. The reaction was terminated by adding 1 mL of 0.5 M CaCl_2_ (Titan Scientific Co., Ltd., Shanghai, China) and 4 mL of 0.5 M NaOH (Titan Scientific Co., Ltd., Shanghai, China), and the released p-nitrophenol was quantified spectrophotometrically at 400 nm. Sucrase activity was assessed by incubating 5 g of soil with 8% sucrose (Titan Scientific Co., Ltd., Shanghai, China) in phosphate buffer (pH 5.5) at 37 °C for 24 h, and the resulting reducing sugars were measured using DNS reagent (Tianjin Aopusheng Chemical Co., Ltd., Tianjin, China) at 508 nm. Urease activity was evaluated by incubating 5 g of soil with 10% urea (Titan Scientific Co., Ltd., Shanghai, China) in citrate buffer (pH 6.7) at 37 °C for 24 h, and the released ammonium was quantified by measuring the indophenol blue complex at 630 nm. For soil chemical properties, air-dried samples sieved through a 2-mm mesh were used with three analytical replicates per parameter. Soil pH and electrical conductivity (EC) were measured in a 1:2.5 (w/v) soil-water suspension (Zhao et al., 2023). Soil organic matter (SOM) was analyzed by the potassium dichromate (Aladdin, Shanghai, China) oxidation method, available phosphorus (AP) was extracted with 0.5 M NaHCO3 (Titan Scientific Co., Ltd., Shanghai, China, pH 8.5) and determined by the molybdenum blue method, alkali-hydrolyzable nitrogen (AN) was assessed by the alkaline hydrolysis diffusion method, and available potassium (AK) was extracted with 1 M ammonium acetate (Titan Scientific Co., Ltd., Shanghai, China, pH 7.0) and measured by flame photometry. All procedures followed established methods (Gao et al., 2023).

### 2.4 Physiological and biochemical indices of leaves

All metabolite data are reported on a fresh weight basis. For antioxidant enzyme analysis, frozen leaf tissue (0.5 g) was homogenized in 5 mL of ice-cold extraction buffer [50 mM phosphate buffer, pH 7.8, containing 1% (w/v) PVP (Titan Scientific Co., Ltd., Shanghai, China) and 0.1 mM EDTA (Aladdin, Shanghai, China)]. After centrifugation (12,000 × g, 20 min, 4 °C), the supernatant was collected for enzymatic assays. Superoxide dismutase (SOD) activity was assayed by monitoring the inhibition of nitroblue tetrazolium (NBT, Aladdin, Shanghai, China) photoreduction at 560 nm. The reaction mixture (3 mL) contained 50 mM phosphate buffer (pH 7.8), 13 mM methionine (Aladdin, Shanghai, China), 75 μM NBT, 0.1 mM EDTA, 2 μM riboflavin (Aladdin, Shanghai, China), and 50 μL of enzyme extract. Following 20 min of illumination, one unit (U) of SOD activity was defined as the amount of enzyme required to achieve 50% inhibition of NBT photoreduction and was expressed as U g^−1^ FW. Catalase (CAT) activity was determined from the decrease in absorbance at 240 nm (ε = 39.4 mM^−1^ cm^−1^) in a 3 mL system containing 50 mM phosphate buffer (pH 7.0), 15 mM H_2_O_2_ (Titan Scientific Co., Ltd., Shanghai, China), and 100 μL of enzyme extract. One unit of CAT activity was defined as the amount of enzyme that decomposes 1 μmol of H_2_O_2_ per minute and was expressed as U g^−1^ FW. Peroxidase (POD) activity was measured by monitoring guaiacol (Titan Scientific Co., Ltd., Shanghai, China) oxidation at 470 nm in a 3 mL mixture containing 50 mM phosphate buffer (pH 7.0), 10 mM H_2_O_2_, 50 mM guaiacol, and 50 μL of enzyme extract. One unit of POD activity was defined as an absorbance increase of 0.01 per minute and was expressed as U g^−1^ FW. Proline content was quantified spectrophotometrically at 520 nm using the acid-ninhydrin (Titan Scientific Co., Ltd., Shanghai, China) method after homogenizing 0.5 g of tissue in 3% sulfosalicylic acid (Titan Scientific Co., Ltd., Shanghai, China), and the concentration was calculated using an L-proline (Titan Scientific Co., Ltd., Shanghai, China) standard curve (μg g^−1^ FW). Malondialdehyde (MDA) concentration was determined by the thiobarbituric acid method, where 0.5 g of tissue was homogenized in 10% trichloroacetic acid (Titan Scientific Co., Ltd., Shanghai, China), reacted with TBA, and the absorbance was measured at 532 and 600 nm. The concentration was calculated using an extinction coefficient of 155 mM^−1^ cm^−1^ (nmol g^−1^ FW). Chlorophyll and carotenoid concentrations were determined by ethanol (Titan Scientific Co., Ltd., Shanghai, China) extraction of 0.2 g of tissue. Absorbance was measured at 665, 649, and 470 nm, and concentrations were calculated according to Lichtenthaler (mg g^−1^ FW). All analyses were conducted with three biological replicates following established methods (Forlani and Funck, 2020; Wu et al., 2019; Zulfiqar and Ashraf, 2023).

### 2.5 Fruit quality

All measurements were performed with three biological replicates. Fresh fruit weight was measured using an analytical balance, and dry weight was determined by oven-drying the fruits at 65 °C until constant weight was achieved. Fruit dimensions, including transverse and longitudinal diameters, were recorded with a vernier caliper. The fruit shape index was calculated as the ratio of longitudinal to transverse diameter, with values closer to 1 indicating a rounder fruit morphology. Fruit firmness was evaluated using a digital hardness tester, while soluble solids content (SSC) was quantified with a handheld refractometer. Soluble sugar content was analyzed by the anthrone-sulfuric acid (Titan Scientific Co., Ltd., Shanghai, China) method (He et al., 2020). Titratable acidity was measured via acid-base titration and expressed as percentage of tartaric acid (Titan Scientific Co., Ltd., Shanghai, China) equivalent (He et al., 2020). Soluble protein content was determined using the Bradford assay (Hussain et al., 2019). Anthocyanin concentration was assessed by the pH differential method (Chongwilaikasem et al., 2024) based on absorbance measurements at 520 nm and 700 nm, and was calculated using the molar extinction coefficient of cyanidin-3-glucoside (Aladdin, Shanghai, China) (ε = 29,600 L·mol^−1^·cm^−1^). Results were expressed as mg cyanidin-3-glucoside equivalent per 1,000 g fresh weight. Vitamin C content was determined by the 2,4-dinitrophenylhydrazine (DNPH, Aladdin, Shanghai, China) colorimetric method (Hussain et al., 2019) and reported as mg per 1,000 g fresh weight (mg/kg FW).

### 2.6 DNA extraction and high-throughput sequencing

Total genomic DNA was extracted from soil microbial communities using the E.Z.N.A.^®^ Soil DNA Kit (Omega Bio-tek, Norcross, GA, USA) following the manufacturer's protocol. DNA quality was assessed via 2% agarose gel electrophoresis (Agarose, BioWest, Shanghai, China), and concentration/purity was measured using a NanoDrop 2000 spectrophotometer (Thermo Scientific, Waltham, MA, USA). The V3–V4 hypervariable regions of the bacterial 16S rRNA gene were amplified with primers 338F(5′-ACTCCTACGGGAGGCAGCAG-3′) and 806R(5′-GGACTACHVGGGTWTCTAAT-3′), while the fungal ITS1 region was amplified using primers ITS1F (5′-CTTGGTCATTTAGAGGAAGTAA-3′) and ITS2R (5′-GCTGCGTTCTTCATCGATGC-3′) (Liu et al., 2016).

PCR amplification was performed in a T100 Thermal Cycler (Bio-Rad, Hercules, CA, USA) under the following conditions: 95 °C for 3 min (pre-denaturation); 27 cycles of 95 °C for 30 s (denaturation), 55 °C for 30 s (annealing), and 72 °C for 45 s (extension); followed by a final extension at 72 °C for 10 min and cooling to 10 °C. Amplified products were purified using a PCR Clean-Up Kit (Yuhua, Changsha, China) after electrophoresis on 2% agarose gels, and quantified with a Qubit 4.0 Fluorometer (Thermo Fisher Scientific, Waltham, MAUSA). Purified amplicons were then processed with the NEXTFLEX Rapid DNA-Seq Kit (PerkinElmer, Waltham, MA, USA) for library preparation and sequenced on the Illumina NextSeq 2000 platform (Shanghai Majorbio Bio-pharm Technology Co., Ltd., China). The raw sequencing data generated in this study have been deposited in the NCBI Sequence Read Archive (SRA) under the BioProject accession number PRJNA1337385.

### 2.7 Bioinformatic analysis

Microbiome data analysis was conducted using QIIME2 (version 2023.2) (Bolyen et al., 2019). For 16S rRNA gene sequences (V3-V4 region), amplicon sequence variants (ASVs) were inferred using the DADA2 pipeline with the following parameters: forward reads were truncated at 280 bp and reverse reads at 220 bp based on quality profiles, with chimeras removed using the consensus method. For fungal ITS1 sequences, reads were trimmed to 250 bp (forward) and 200 bp (reverse) before DADA2 denoising. Taxonomic classification of ASVs was performed using the Naïve Bayes classifier in QIIME2 with region-specific reference databases: bacterial sequences were classified using a SILVA v138 classifier trained specifically for the V3-V4 region, while fungal sequences were classified with a UNITE v8.0 classifier specific to the ITS1 locus.

Venn diagrams were constructed using Python 3.10.12 to visualize shared and unique ASVs across treatments. Alpha diversity indices (Chao1, Shannon) were calculated using mothur (version 1.48.0) (Schloss et al., 2009). Beta diversity was assessed using unweighted UniFrac for the bacterial community and the abund-jaccard index for the fungal community, respectively. Prior to this analysis, the bacterial and fungal datasets were independently rarefied to standardize sequencing effort. The bacterial data were rarefied to a depth of 38,278 sequences per sample, while the fungal data were rarefied to 22,075 sequences per sample, with all original samples (100%) retained in both cases. Principal coordinates analysis (PCoA) was used to visualize community separation, and permutational multivariate analysis of variance (PERMANOVA) with 999 permutations was performed using the adonis function in the vegan package implemented in R (version 4.3.2) to test for significant differences among treatments.

Microbial community composition and co-occurrence networks were analyzed in R (version 4.3.2) using the vegan and igraph packages, respectively. Microbial associations were inferred by calculating Spearman correlation coefficients using R-4.3.2 with support from Python-3.10.12, with an absolute correlation threshold of 0.6 and significance level of *p* < 0.05. Multiple testing correction was applied using the Benjamini-Hochberg false discovery rate (FDR) procedure. To statistically identify connector taxa, we implemented a null model approach using the Maslov-Sneppen algorithm with 1,000 randomizations; taxa were considered statistically significant connectors if their betweenness centrality exceeded the 95th percentile (*p* < 0.05) of the null distribution and their original betweenness centrality was >75th percentile while their degree centrality was < 25th percentile within the observed network. For correlations with soil properties, Spearman's rank correlation was performed focusing on the top 50 most abundant genera, which collectively represented >50% of total sequences, thus capturing the most ecologically influential community members while reducing noise from rare taxa. Network visualization was optimized using Gephi (version 0.10.1), where node centrality and edge weights were analyzed to identify these connector taxa. All the aforementioned bioinformatic analyses, including initial data processing and statistical computations, were performed using the Majorbio Cloud Platform (www.majorbio.com).

### 2.8 Statistical analysis

All statistical analyses were conducted using IBM SPSS Statistics (Version 25.0). The study was designed with three independent replicates per treatment group, and results are expressed as the mean ± standard deviation. The assumptions of ANOVA were verified by testing the normality of residuals using the Shapiro–Wilk test and assessing the homogeneity of variances with Levene's test. For all datasets where the one-way ANOVA revealed a significant effect, Tukey's honestly significant difference (HSD) test was employed for *post-hoc* pairwise comparisons. A p-value of less than 0.05 was deemed statistically significant. The microbial community analysis was implemented with R (version 4.3.2). Specifically, differential abundance testing was performed using the non-parametric Kruskal–Wallis test. In accordance with standard practice for non-parametric testing, *post-hoc* pairwise comparisons were conducted using Dunn's test, with p-values corrected for multiple comparisons via the Benjamini–Hochberg FDR method. Statistical significance for these tests was defined at an FDR-corrected threshold of *p*-_adj_ < 0.05. This comprehensive statistical evaluation was applied to all quantitative data generated in this study.

## 3 Results

### 3.1 Effects of different inoculant application rates on grape rhizosphere soil physicochemical properties

**Figure 1:**
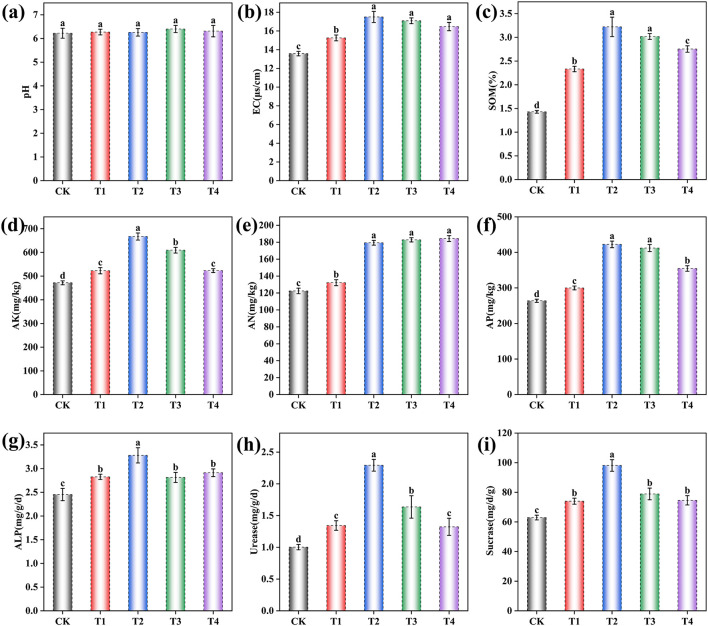
Effects of different inoculant application rates on grape rhizosphere soil physicochemical properties. **(a)** pH; **(b)** Electrical conductivity (EC); **(c)** Soil organic matter (SOM); **(d)** Available potassium (AK); **(e)** Available nitrogen (AN); **(f)** Available phosphorus (AP); **(g)** Alkaline phosphatase (ALP) activity; **(h)** Urease activity; **(i)** Sucrase activity. CK (no inoculant treatment), T1 (45 L/ha inoculant), T2 (90 L/ha inoculant), T3 (135 L/ha inoculant), T4 (180 L/ha inoculant). Bars and lines represent mean values of three replicates ± SE (standard error). A different letter at the head of a column indicates a significant difference (*p* < 0.05) from other treatments. All statistical analyses were based on three biological replicates (*n* = 3) per treatment.

The application of the microbial inoculant significantly modified grape rhizosphere soil properties, although no significant effect on pH was observed [*F* (4, 10) = 0.80, *p* = 0.552] (Figure 1 and Supplementary Table S2). One-way ANOVA revealed significant treatment effects on all other soil physicochemical properties and enzyme activities (*p* < 0.001 for all; Supplementary Table S3). Subsequent Tukey's HSD *post-hoc* tests showed that soil electrical conductivity (EC) was significantly higher in all inoculant-treated groups [*F* (4, 10) = 85.64, *p* < 0.001], increasing by 12.37% in T1, 28.87% in T2, 25.85% in T3, and 21.32% in T4 compared to the CK (Figure 1b). Similarly, soil organic matter (SOM) content significantly rose [*F* (4, 10) = 225.97, *p* < 0.001] by 63.21% in T1, 125.32% in T2, 111.15% in T3, and 92.63% in T4 (Figure 1c). The available nitrogen (AN) significantly increased [*F* (4, 10) = 279.49, *p* < 0.001] by 7.96% in T1, 46.44% in T2, 49.32% in T3, and 50.57% in T4 (Figure 1d), while available potassium (AK) showed significant enhancement [*F* (4, 10) = 136.18, *p* < 0.001] by 10.81% in T1, 41.41% in T2, 29.19% in T3, and 10.88% in T4 (Figure 1e). Available phosphorus (AP) demonstrated significant increases [*F* (4, 10) = 244.62, *p* < 0.001] of 13.71% in T1, 60.13% in T2, 56.27% in T3, and 34.45% in T4 (Figure 1f). Furthermore, soil enzyme activities were significantly enhanced in all treatments compared to the CK. Alkaline phosphatase (ALP) activity increased by 15.22% in T1, 33.70% in T2, 14.67% in T3, and 18.75% in T4 [*F* (4, 10) = 20.73, *p* < 0.001; Figure 1g]; urease activity rose by 33.89% in T1, 128.57% in T2, 63.12% in T3, and 31.89% in T4 [*F* (4, 10) = 14.52, *p* < 0.001; Figure 1h]; and sucrase activity augmented by 17.56% in T1, 55.88% in T2, 25.34% in T3, and 18.50% in T4 [*F* (4, 10) = 189.04, *p* < 0.001; Figure 1i].

### 3.2 Effects of different inoculant application rates on physiological and biochemical indices of grape leaves

**Figure 2:**
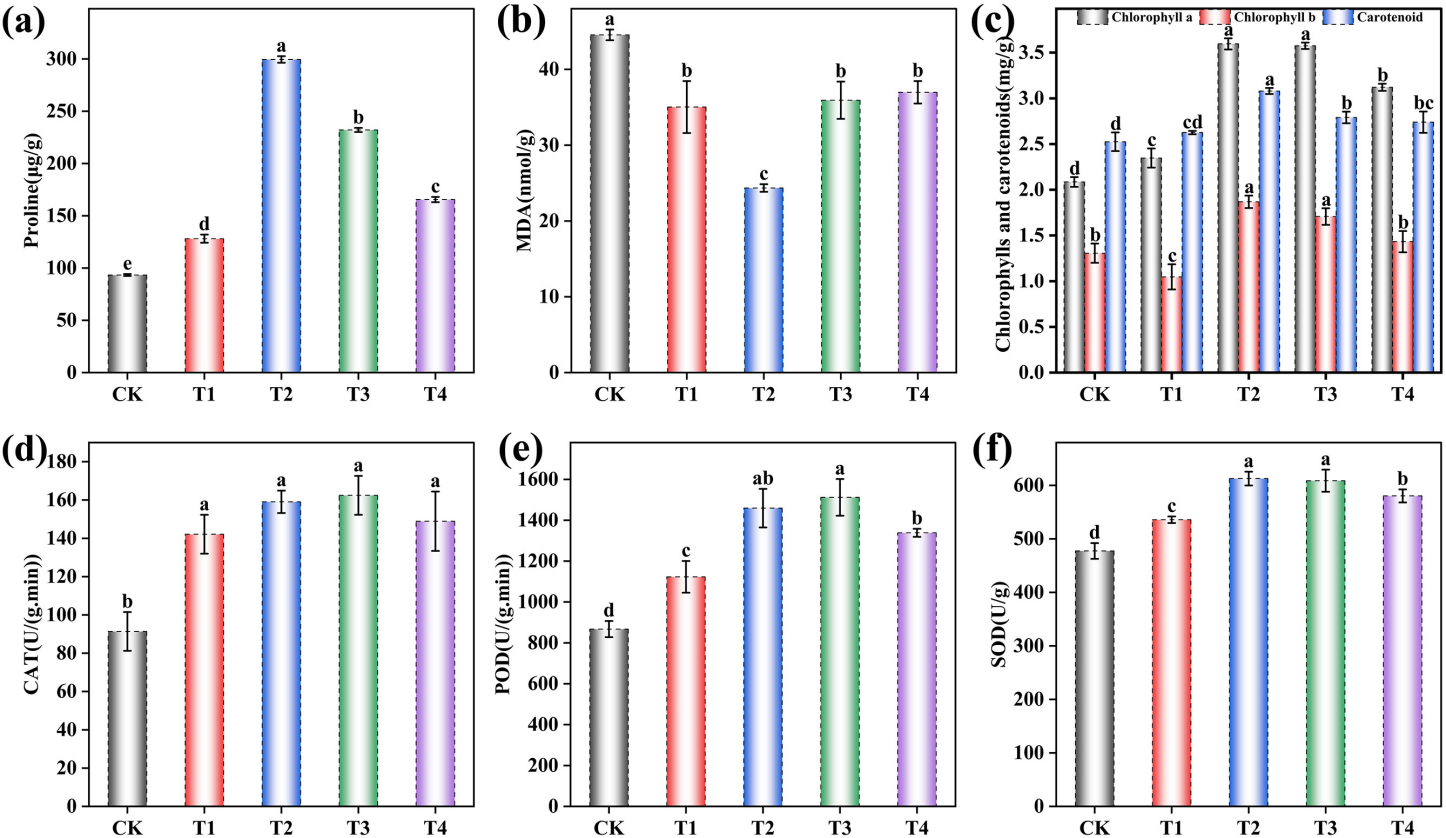
Effects of different inoculant application rates on grape leaf physiological and biochemical indices. **(a)** Proline content; **(b)** Malondialdehyde (MDA) content; **(c)** Chlorophyll content; **(d)** Catalase (CAT) activity; **(e)** Peroxidase (POD) activity; **(f)** Superoxide dismutase (SOD) activity. CK (no inoculant treatment), T1 (45 L/ha inoculant), T2 (90 L/ha inoculant), T3 (135 L/ha inoculant), T4 (180 L/ha inoculant). Bars and lines represent mean values of three replicates ± SE (standard error). A different letter at the head of a column indicates a significant difference (*p* < 0.05) from other treatments. All statistical analyses were based on three biological replicates (*n* = 3) per treatment.

The physiological and biochemical parameters of grape leaves under different microbial inoculant application rates were systematically evaluated (Figure 2 and Supplementary Table S4). One-way ANOVA revealed highly significant treatment effects on all measured parameters (*p* < 0.001).

Proline content was significantly influenced by inoculant application [*F* (4, 10) = 2837.73, *p* < 0.001], increasing by 37.21% in T1, 220.87% in T2, 148.58% in T3, and 77.29% in T4 compared to CK (Figure 2a). Malondialdehyde (MDA) content showed a significant decrease [*F* (4, 10) = 359.69, *p* < 0.001] of 21.37% in T1, 45.35% in T2, 19.35% in T3, and 17.01% in T4 (Figure 2b). Photosynthetic pigments were significantly affected by the treatments. Chlorophyll a content increased substantially [*F* (4, 10) = 28.02, *p* < 0.001] by 12.56% in T1, 72.44% in T2, 71.43% in T3, and 49.66% in T4. Chlorophyll b content was also significantly altered [*F* (4, 10) = 655.23, *p* < 0.001], decreasing by 19.82% in T1 while increasing by 43.05% in T2, 30.70% in T3, and 9.69% in T4. Carotenoid content showed significant enhancement [*F* (4, 10) = 22.50, *p* < 0.001] with increases of 3.99% in T1, 21.99% in T2, 10.52% in T3, and 8.49% in T4 (Figure 2c). Antioxidant enzyme activities were markedly enhanced by the inoculant treatments. Catalase (CAT) activity increased significantly [*F* (4, 10) = 21.27, *p* < 0.001] by 55.55% in T1, 74.07% in T2, 77.77% in T3, and 62.96% in T4. Peroxidase (POD) activity showed substantial improvement [*F* (4, 10) = 42.40, *p* < 0.001] with increases of 29.46% in T1, 68.19% in T2, 74.28% in T3, and 54.30% in T4. Superoxide dismutase (SOD) activity was significantly elevated [*F* (4, 10) = 48.74, *p* < 0.001] by 12.22% in T1, 28.35% in T2, 27.50% in T3, and 21.56% in T4 (Figures 2d–f). Complete ANOVA results for all physiological and biochemical parameters are provided in Supplementary Table S5.

### 3.3 Effects of different inoculant application rates on grape fruit quality

**Figure 3:**
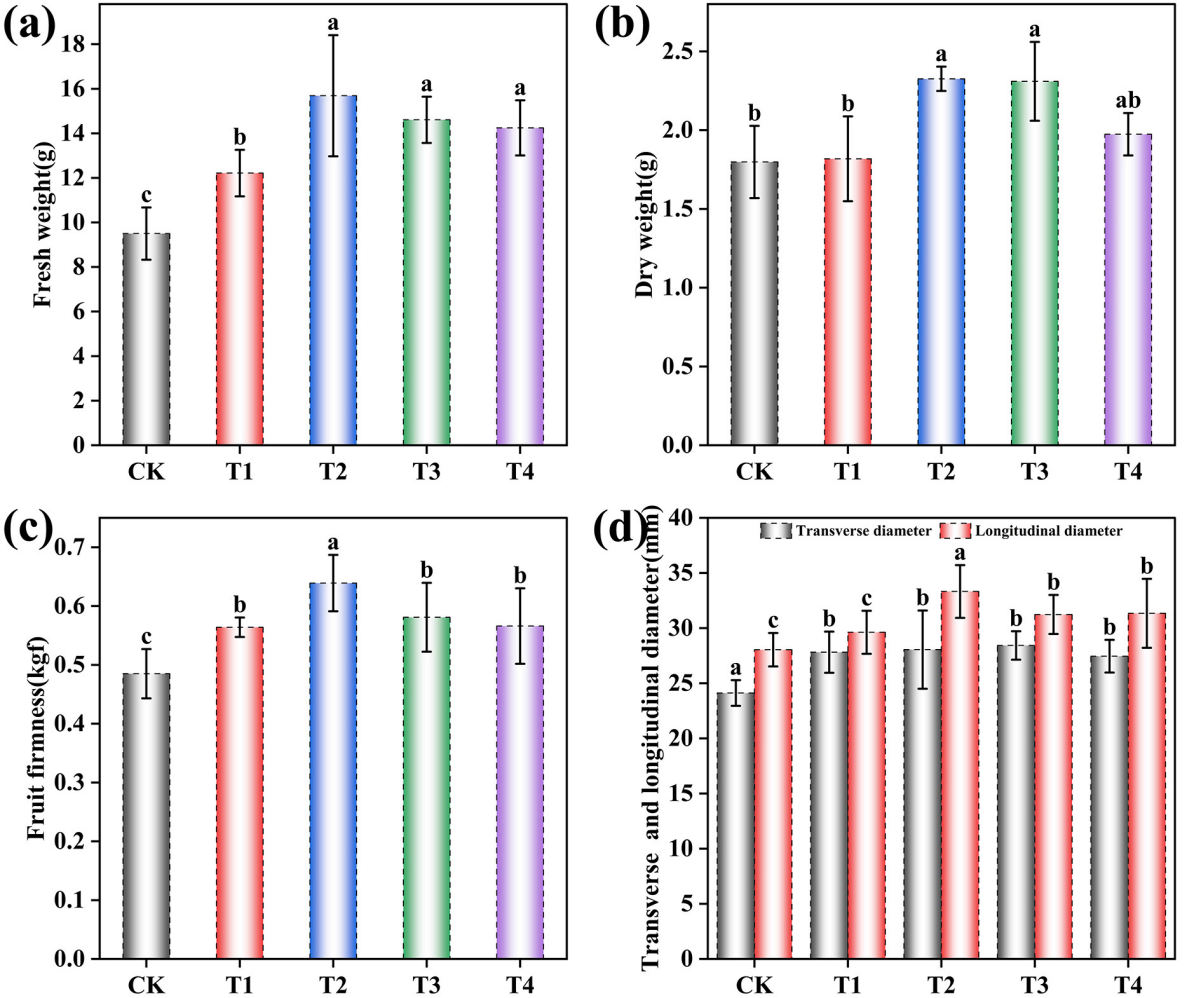
Effects of different inoculant application rates on the apparent quality of grape fruits. **(a)** Fresh weight per fruit; **(b)** Dry weight per fruit; **(c)** Fruit firmness; **(d)** Fruit transverse diameter and longitudinal diameter. CK (no inoculant treatment), T1 (45 L/ha inoculant), T2 (90 L/ha inoculant), T3 (135 L/ha inoculant), T4 (180 L/ha inoculant). Bars and lines represent mean values of three replicates ± SE (standard error). A different letter at the head of a column indicates a significant difference (*p* < 0.05) from other treatments. All statistical analyses were based on three biological replicates (*n* = 20) per treatment.

The present study demonstrated that the application of the compound microbial inoculant significantly enhanced both the visual and nutritional quality of grape fruits (Figure 3, Supplementary Table S6, and Table 1). One-way ANOVA revealed highly significant treatment effects on all measured physical parameters (*p* < 0.001).

**Table 1:** Effects of different inoculant application rates on the intrinsic nutritional quality of grape fruits.

**Treatment**	**Titratable acidity (%)**	**Soluble sugar (%)**	**Sugar-acid ratio**	**Soluble sugar (%)**	**Soluble protein (mg/kg FW)**	**Anthocyanins (mg/kg FW)**	**Vitamin C (mg/kg FW)**
CK	0.53 ± 0.01a	14.96 ± 0.85c	28.46 ± 1.00d	16.12 ± 0.77cd	21.27 ± 2.12c	6.25 ± 0.64c	7.84 ± 0.38d
T1	0.50 ± 0.02b	15.66 ± 0.23bc	31.66 ± 1.36c	17.80 ± 0.40c	37.24 ± 2.44b	6.55 ± 0.44c	11.27 ± 0.57c
T2	0.44 ± 0.01c	19.17 ± 0.57a	43.22 ± 1.13a	21.70 ± 1.27a	52.74 ± 6.30a	8.60 ± 0.30ab	16.01 ± 0.32a
T3	0.45 ± 0.01c	18.22 ± 0.41a	40.50 ± 0.92ab	21.25 ± 1.14a	56.45 ± 4.17a	8.91 ± 0.15a	16.20 ± 0.91a
T4	0.45 ± 0.01c	18.15 ± 0.52a	40.03 ± 1.39b	19.80 ± 1.04b	43.95 ± 3.87b	7.68 ± 0.15b	14.29 ± 0.35b

CK (no inoculant treatment), T1 (45 L/ha inoculant), T2 (90 L/ha inoculant), T3 (135 L/ha inoculant), T4 (180 L/ha inoculant). Different lowercase letters represent significant differences between different treatments in the same column. All statistical analyses were based on three biological replicates (*n* = 3) per treatment.

The T1-T4 treatments resulted in marked improvements across multiple physical parameters: fresh weight per fruit showed significant enhancement [*F* (4, 95) = 51.43, *p* < 0.001], increasing by 28.57% in T1, 65.13% in T2, 53.75% in T3, and 49.95% in T4 compared to CK (Figure 3a); dry weight per fruit was significantly affected [*F* (4, 95) = 49.78, *p* < 0.001], rising by 1.11% in T1, 29.37% in T2, 28.48% in T3, and 9.79% in T4 (Figure 3b); fruit firmness was significantly enhanced [*F* (4, 95) = 44.32, *p* < 0.001] by 16.29% in T1, 31.75% in T2, 19.79% in T3, and 16.70% in T4 (Figure 3c); transverse diameter increased significantly [*F* (4, 95) = 19.80, *p* < 0.001] by 15.31% in T1, 16.29% in T2, 17.86% in T3, and 13.82% in T4; and longitudinal diameter showed significant growth [*F* (4, 95) = 178.62, *p* < 0.001] by 5.65% in T1, 18.84% in T2, 11.40% in T3, and 11.77% in T4 (Figure 3d). The fruit shape index approached 1 across all treatments, indicating a consistent near-spherical morphology.

With regard to nutritional quality, all parameters showed highly significant treatment effects (*p* < 0.001). Titratable acidity decreased significantly [*F* (4, 10) = 31.87, *p* < 0.001] by 5.77% in T1, 15.55% in T2, 14.34% in T3, and 13.71% in T4, whereas soluble sugar content increased significantly [*F* (4, 10) = 32.55, *p* < 0.001] by 4.66% in T1, 28.15% in T2, 21.80% in T3, and 21.28% in T4, resulting in elevated sugar-to-acid ratios [*F* (4, 10) = 87.47, *p* < 0.001] of 11.22%, 51.84%, 42.29%, and 40.64%, respectively. Additionally, soluble solids content improved significantly [*F* (4, 10) = 27.49, *p* < 0.001] by 10.43% in T1, 34.62% in T2, 31.87% in T3, and 22.83% in T4; soluble protein content increased significantly [*F* (4, 10) = 35.48, *p* < 0.001] by 75.08% in T1, 148.00% in T2, 165.40% in T3, and 106.63% in T4; anthocyanin content rose significantly [*F* (4, 10) = 28.65, *p* < 0.001] by 4.80% in T1, 37.47% in T2, 42.54% in T3, and 22.76% in T4; and vitamin C content was significantly enhanced [*F* (4, 10) = 124.05, *p* < 0.001] by 43.70% in T1, 104.12% in T2, 106.48% in T3, and 82.13% in T4. Complete ANOVA results for all fruit quality parameters are provided in Supplementary Table S7.

### 3.4 ASV analysis and alpha diversity of grape rhizosphere soil microbiota under different inoculant application rates

**Figure 4:**
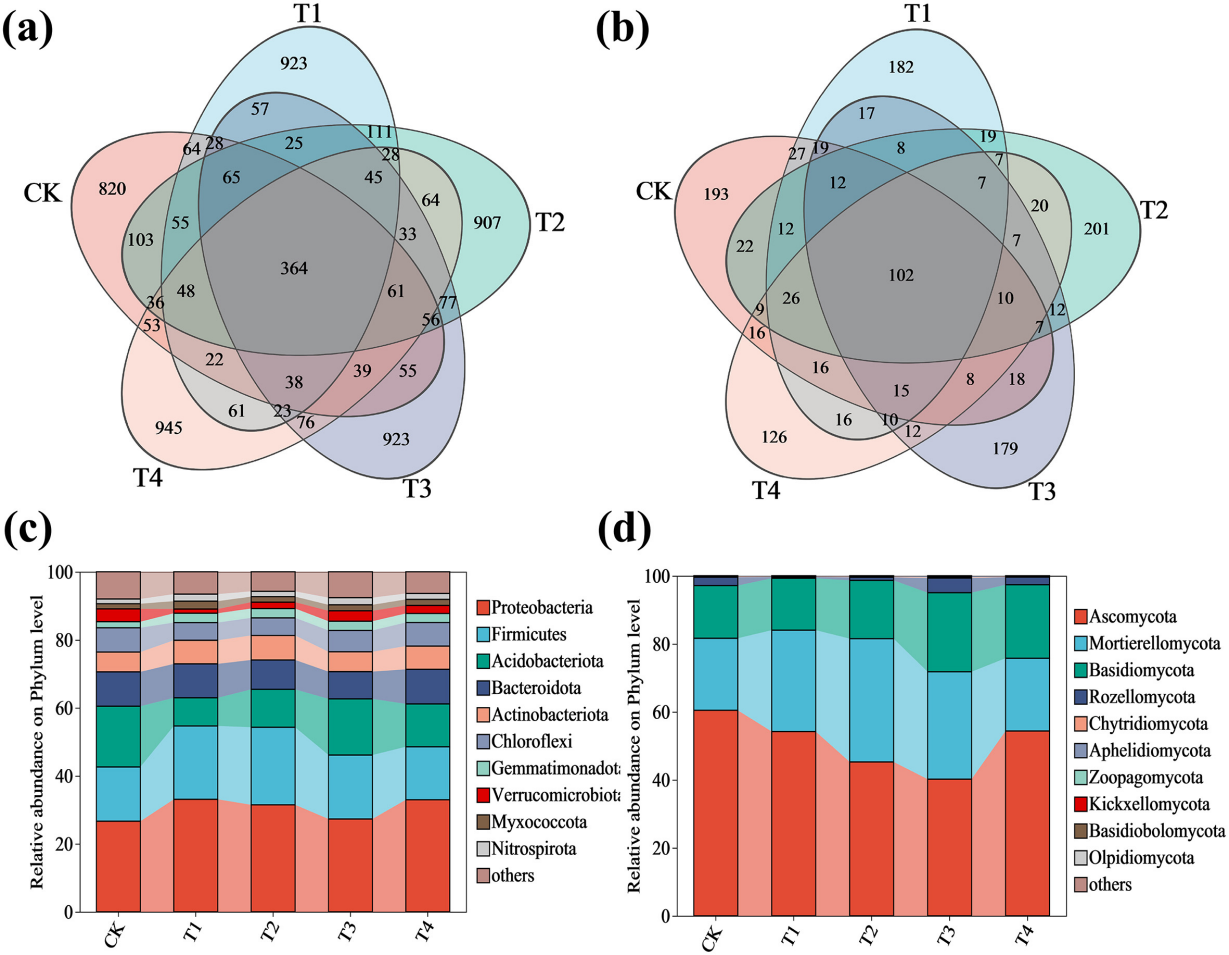
Microbial community composition and distribution in grape rhizosphere soil under inoculant application rates. **(a)** Venn diagram of bacterial ASVs. **(b)** Venn diagram of fungal ASVs. **(c)** Phylum-level relative abundance of bacterial communities. **(d)** Phylum-level relative abundance of fungal communities. CK (no inoculant treatment), T1 (45 L/ha inoculant), T2 (90 L/ha inoculant), T3 (135 L/ha inoculant), T4 (180 L/ha inoculant). The diagrams show the distribution of ASVs, with overlapping regions indicating shared ASVs among treatments and non - overlapping regions representing ASVs unique to specific treatments.

The microbial community structure and diversity in grape rhizosphere soil under different fertilization treatments were evaluated using amplicon sequence variant (ASV) analysis and alpha diversity indices (Figure 4 and Table 2). After quality filtering and chimera removal, a total of 636,129 high-quality sequences were obtained from the 15 samples, with an average of 42,409 ± 3,556 sequences per sample. Venn diagram analysis revealed a core microbiome of 364 bacterial and 102 fungal ASVs that were common to all groups, including the control (CK). More importantly, we identified 45 bacterial and 7 fungal ASVs that were uniquely shared among the four inoculant treatments (T1–T4) but were absent in the CK group, highlighting a specific microbial signature induced by the application of the microbial inoculant. The number of treatment-specific ASVs was 820 (CK), 923 (T1), 907 (T2), 923 (T3), and 945 (T4) for bacteria, and 193 (CK), 182 (T1), 201 (T2), 179 (T3), and 126 (T4) for fungi (Figures 4a, b).

**Table 2:** Bacterial and fungal alpha diversity indices of grape rhizosphere soil under different inoculant application rates.

	**Treatment**	**Sobs**	**ACE**	**Chao 1**	**Shannon**	**Simpson**	**Pielou_e**	**Coverage (%)**
Bacteria	CK	1,907 ± 66b	1,915.98 ± 69b	1,908.84 ± 66b	6.8522 ± 0.14a	0.0019 ± 0.0002a	0.91 ± 0.01a	1.00
	T1	1,957 ± 81ab	1,962.59 ± 88ab	1,957.79 ± 81ab	6.9287 ± 0.25a	0.0019 ± 0.0002a	0.91 ± 0.01a	1.00
	T2	2,078 ± 71a	2,089.35 ± 76a	2,081.56 ± 74a	6.9301 ± 0.21a	0.0019 ± 0.0003a	0.91 ± 0.01a	1.00
	T3	1,965 ± 49ab	1,980.40 ± 55ab	1,969.35 ± 53ab	6.8425 ± 0.11a	0.0021 ± 0.0001a	0.90 ± 0.03a	1.00
	T4	1,936 ± 58b	1,936.00 ± 58b	1,936.00 ± 58b	6.8565 ± 0.35a	0.0020 ± 0.0001a	0.91 ± 0.02a	1.00
Fungi	CK	512 ± 45a	514.16 ± 46a	512.70 ± 45a	4.5163 ± 0.13a	0.0278 ± 0.0007d	0.72 ± 0.02a	1.00
	T1	495 ± 48a	497.22 ± 49a	495.70 ± 48a	4.4950 ± 0.09a	0.0241 ± 0.0015e	0.72 ± 0.01a	1.00
	T2	481 ± 35ab	481.00 ± 35ab	481.00 ± 35ab	4.1051 ± 0.11b	0.0427 ± 0.0009c	0.66 ± 0.02b	1.00
	T3	443 ± 40ab	443.00 ± 40ab	443.00 ± 40ab	3.9978 ± 0.18b	0.0505 ± 0.0013a	0.66 ± 0.02b	1.00
	T4	407 ± 37b	407.00 ± 37b	407.00 ± 37b	3.9418 ± 0.21b	0.0471 ± 0.0010b	0.66 ± 0.03b	1.00

CK (no inoculant treatment), T1 (45 L/ha inoculant), T2 (90 L/ha inoculant), T3 (135 L/ha inoculant), T4 (180 L/ha inoculant). Different lowercase letters represent significant differences between different treatments in the same column. Indices include: Sobs (observed richness); ACE (abundance-based coverage estimator); Chao1 (Chao1 richness estimator); Shannon (Shannon diversity index); Simpson (Simpson diversity index); Pielou_e (Pielou's evenness index); Coverage (Good's coverage).

Alpha diversity was assessed using established indices with statistical validation (Table 2 and Supplementary Table S8). The Goods coverage index approached 1.0 for both bacterial and fungal communities, confirming adequate sequencing depth. One-way ANOVA revealed significant treatment effects on bacterial richness indices: Sobs [*F* (4, 10) = 8.67, *p* = 0.003), ACE [*F* (4, 10) = 8.43, *p* = 0.003), and Chao1 [*F* (4, 10) = 8.83, *p* = 0.003), with Tukey's HSD *post-hoc* test confirming elevated richness in T1-T4 compared to CK. In contrast, fungal richness indices showed no significant differences (Sobs: *F* (4, 9) = 3.42, *p* = 0.058; ACE: *F* (4, 9) = 3.39, *p* = 0.059; Chao1: *F* (4, 9) = 3.41, *p* = 0.058). Bacterial diversity (Shannon: *F* (4, 10) = 0.58, *p* = 0.684; Simpson: *F* (4, 10) = 2.40, *p* = 0.119) and evenness (Pielou_e: *F* (4, 10) = 0.38, *p* = 0.821) remained stable across treatments. However, fungal communities showed significantly reduced diversity (Shannon: *F* (4, 9) = 4.88, *p* = 0.023; Simpson: *F* (4, 9) = 10.74, *p* = 0.002) and evenness (Pielou_e: *F* (4, 9) = 4.44, *p* = 0.030) under higher inoculant application rates. These results demonstrate that inoculant application differentially influences bacterial and fungal community structures, enhancing bacterial richness while reducing fungal diversity and evenness.

### 3.5 Bacterial and fungal community structures under different inoculant application rates

The application of compound microbial inoculants significantly altered both bacterial and fungal community structures at the phylum level in grape rhizosphere soil. One-way ANOVA revealed significant treatment effects on the relative abundance of all major bacterial and fungal phyla (*p* < 0.001 for all).

For bacterial communities (Figure 4c), treatments T1–T4 resulted in the following statistically significant changes compared to the control (CK): the relative abundance of Proteobacteria showed significant variation [*F* (4, 10) = 11.27, *p* = 0.001), increasing by 24.22% in T1, 18.21% in T2, 2.52% in T3, and 23.77% in T4; Firmicutes abundance was significantly affected [*F* (4, 10) = 44.28, *p* < 0.001), increasing by 35.19% in T1, 42.83% in T2, and 17.78% in T3, with no significant change in T4; Actinobacteriota demonstrated significant changes [*F* (4, 10) = 24.13, *p* < 0.001), increasing by 19.04% in T1, 23.67% in T2, 0.34% in T3, and 17.15% in T4; Gemmatimonadota showed significant enhancement [*F* (4, 10) = 21.18, *p* < 0.001), increasing by 54.80% in T1, 55.37% in T2, 53.11% in T3, and 50.28% in T4; Myxococcota abundance was significantly altered [*F* (4, 10) = 39.82, *p* < 0.001), increasing by 51.97% in T1, 6.58% in T2, 16.45% in T3, and 16.45% in T4; and Nitrospirota showed significant increases [*F* (4, 10) = 49.20, *p* < 0.001), rising by 49.64% in T1, 12.95% in T2, 49.64% in T3, and 26.62% in T4. Conversely, the relative abundance of Acidobacteriota decreased significantly [*F* (4, 10) = 38.03, *p* < 0.001) by 53.64% in T1, 37.63% in T2, 7.45% in T3, and 29.34% in T4; Bacteroidota showed significant reduction [*F* (4, 10) = 124.93, *p* < 0.001) by 14.46% in T2 and 20.79% in T3; Chloroflexi decreased significantly [*F* (4, 10) = 74.85, *p* < 0.001) by 27.13% in T1, 27.55% in T2, 12.31% in T3, and 2.94% in T4; and Verrucomicrobiota abundance was significantly reduced [*F* (4, 10) = 181.89, *p* < 0.001) by 67.28% in T1, 50.66% in T2, 19.53% in T3, and 37.73% in T4 (Supplementary Figure S1). For fungal communities (Figure 4d), the relative abundance of Ascomycota decreased significantly [*F* (4, 10) = 35.91, *p* < 0.001) by 10.34% in T1, 25.12% in T2, 33.52% in T3, and 10.06% in T4. In contrast, Mortierellomycota increased significantly [*F* (4, 10) = 47.23, *p* < 0.001) by 40.73% in T1, 71.07% in T2, 49.08% in T3, and 0.94% in T4, while Basidiomycota showed significant enhancement [*F* (4, 10) = 70.00, *p* < 0.001] by 10.51% in T2, 50.03% in T3, and 39.39% in T4 (Supplementary Figure S2). Complete ANOVA results for all phylum-level taxa are provided in Supplementary Table S9.

**Figure 5:**
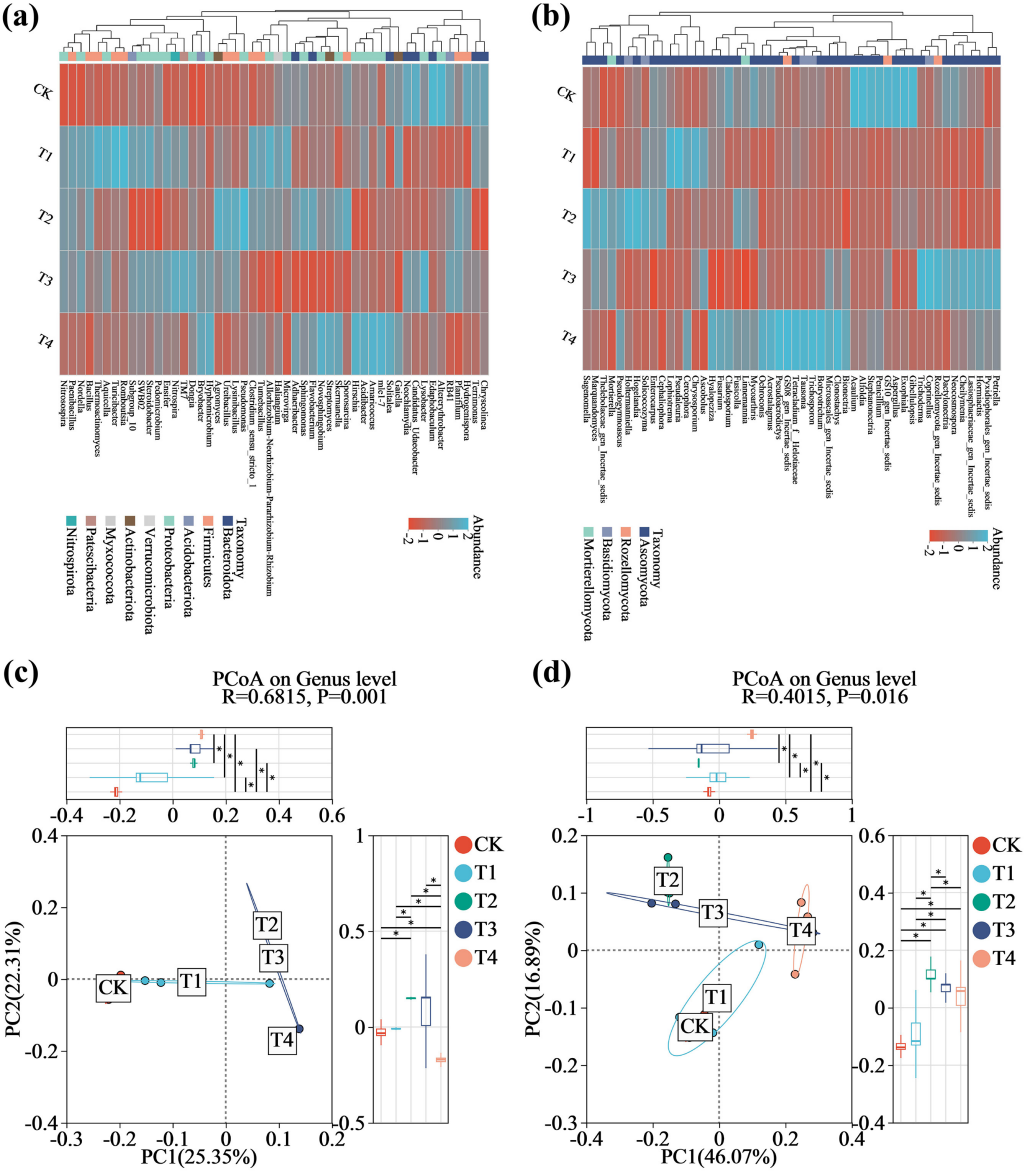
Differences in microbial community structure in grape rhizosphere soil across inoculant application rates. **(a)** Heatmap of the top 50 bacterial genera. **(b)** Heatmap of the top 50 fungal genera. **(c)** Principal coordinates analysis of bacterial communities. **(d)** Principal coordinates analysis of fungal communities. Blue and red colors in panels **(a)** and **(b)** denote higher and lower relative abundance, respectively. CK (no inoculant treatment), T1 (45 L/ha inoculant), T2 (90 L/ha inoculant), T3 (135 L/ha inoculant), T4 (180 L/ha inoculant).

Comparative analysis of bacterial and fungal community compositions at the genus level across the five soil treatments revealed substantial shifts in dominant genera and their relative abundances (Figure 5). Kruskal-Wallis rank-sum tests with FDR adjustment identified several taxa showing statistically significant abundance changes (*p-*_adj_ < 0.05). In bacterial communities, the application of the compound microbial inoculant resulted in the significant enrichment of beneficial taxa including *Bacillus* (H = 9.67, *p* = 0.022, *p*_−adj_ = 0.039), *Nitrospira* (H = 9.70, *p* = 0.021, *p*_−adj_ = 0.039), *Pseudomonas* (H = 10.42, *p* = 0.015, *p*_−adj_ = 0.031), *Flavobacterium* (H = 9.97, *p* = 0.019, *p*_−adj_ = 0.039), *Bryobacter* (H = 9.50, *p* = 0.023, *p*_−adj_ = 0.039), and *Planifilum* (H = 9.70, *p* = 0.021, *p*_−adj_ = 0.039). Notably, the relative abundances of *Bacillus* and *Pseudomonas* were elevated in T1 and T2 but stabilized in T3 and T4, suggesting a dose-dependent inhibitory effect. In contrast, genera including *Sphingomonas* (H = 9.97, *p* = 0.019, *p*_−adj_ = 0.039) and *RB41* (H = 8.23, *p* = 0.041, *p*_−adj_ = 0.048) were significantly reduced across all treatment groups (Figure 5a and Supplementary Figure S3a). Within fungal communities, taxa such as *Tausonia* (H = 11.13, *p* = 0.025, *p*_−adj_ = 0.036), *Linnemannia* (H = 11.57, *p* = 0.021, *p*_−adj_ = 0.036), *Penicillium* (H = 13.06, *p* = 0.011, *p*_−adj_ = 0.024), *Hormiactis* (H = 11.43, *p* = 0.022, *p*_−adj_ = 0.036), and *Fusarium* (H = 10.51, *p* = 0.033, *p*_−adj_ = 0.045) exhibited significantly reduced relative abundances in T1-T4, whereas *Mortierella* (H = 10.30, *p* = 0.036, *p*_−adj_ = 0.045), *Cephaliophora* (H = 10.66, *p* = 0.013, *p*_−adj_ = 0.045), *Solicoccozyma* (H = 11.03, *p* = 0.026, *p*_−adj_ = 0.036), and *Pseudaleuria* (H = 11.13, *p* = 0.025, *p*_−adj_ = 0.036) demonstrated marked and statistically significant enrichment under inoculant application (Figure 5b and Supplementary Figure S3b). Complete Kruskal-Wallis test results for all bacterial and fungal genera are provided in Supplementary Table S10.

Principal Coordinates Analysis (PCoA) revealed significant restructuring of bacterial and fungal β-diversity among microbial inoculant treatments (Figures 5c, d). ANOSIM further confirmed significant inter-treatment differences (bacteria: *R* = 0.6815, *p* = 0.001; fungi: *R* = 0.4015, *p* = 0.016). For bacterial communities (Figure 5c), PC1 (explaining 25.35% of the variance) showed significant separation between treatment T3 and the control (CK) (*p* < 0.05), while partial overlap between T1 and CK indicated 1′s relatively minor impact on community structure. Strikingly, T2 showed distinct separation from T1 and T4 but clustered with T3 along PC1. Along PC2 (22.31% of variance), T2 was further distinguished from other treatments (*p* < 0.05). Principal component analysis revealed distinct patterns in fungal community composition (Figure 5d). PC1 (explaining 46.07% of the variance) revealed clear separation between treatment groups T2 through T4 and the control (CK), with T2 being completely distinct from T1, T3, and T4, while partial overlap was observed between T1 and CK (*p* < 0.05). Moreover, PC2 (explaining 16.89% of the variance) further demonstrated significant separation of T2 from the other treatment groups (*p* < 0.05).

### 3.6 Network analysis of microbial co-occurrence and its correlation with soil physicochemical properties

**Figure 6:**
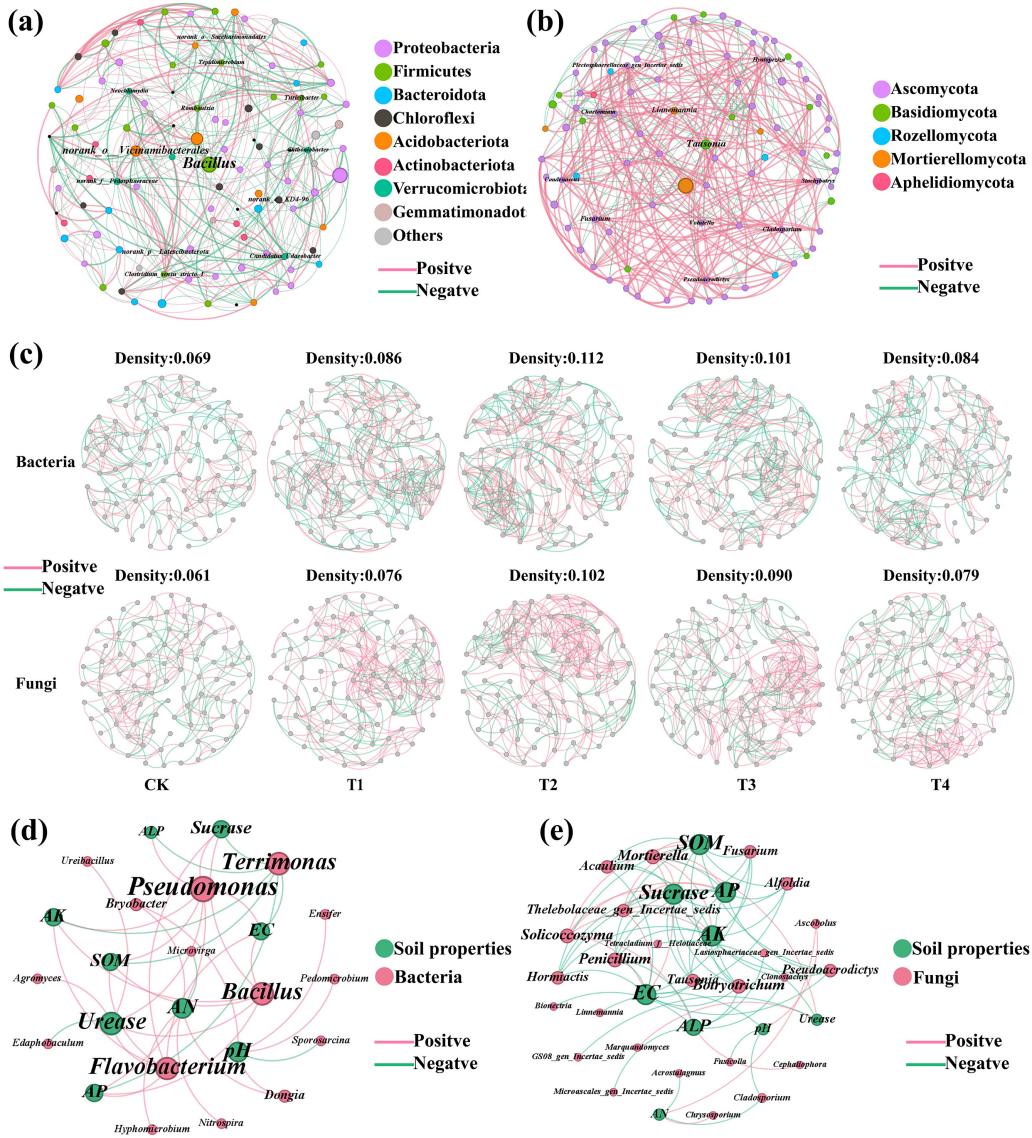
Network analysis of soil microbial co-occurrence and microbial-soil property correlations. **(a)** Bacterial co-occurrence network. **(b)** Fungal co-occurrence network. **(c)** Bacterial and fungal network analysis for each treatment: CK (no inoculant treatment), T1 (45 L/ha inoculant), T2 (90 L/ha inoculant), T3 (135 L/ha inoculant), T4 (180 L/ha inoculant). **(d)** Correlation network between bacterial genera and soil physicochemical properties. **(e)** Correlation network between fungal genera and soil physicochemical properties. Connections are based on significant correlations (*r* > 0.6, *p* < 0.05).

Network analysis was performed on the top 100 bacterial and fungal genera ranked by relative abundance. The bacterial co-occurrence network contained 100 nodes and 437 edges, exhibiting an approximately equal proportion of positive and negative correlations (50.34% vs. 49.66%; Figure 6a). At the phylum level, the taxonomic composition was characterized as follows: Proteobacteria (32.31%), Firmicutes (17.13%), Bacteroidota (10.11%), Chloroflexi (9.18%), Acidobacteriota (8.06%), Actinobacteriota (6.01%), Verrucomicrobiota (5.10%), and Gemmatimonadota (3.15%). The connector genus *Bacillus* exhibited significant positive correlations with several genera, including *Paenibacillus, Pseudomonas, Nitrosospira, Tepidimicrobium, Agromyces, Ureibacillus*, and *Oceanobacillus*. Other taxa that served as network hubs included *Romboutsia, Candidatus Udaeobacter, Neochlamydia, Turicibacter, Tepidimicrobium*, and *Chthoniobacter* (Figure 6a and Supplementary Tables S11, S12). The fungal co-occurrence network consisted of 95 nodes and 383 edges, and was characterized by a predominance of positive correlations (65.34% positive vs. 34.66% negative; Figure 6b). In terms of taxonomic composition, the network was primarily composed of Ascomycota (76.60%), Mortierellomycota (13.83%), Rozellomycota (4.26%), Basidiomycota (4.26%), and Aphelidiomycota (1.06%). Hub taxa, including *Chaetotium, Condenascus*, and *Volutella*, demonstrated the highest degree of connectivity within the network. Among these, *Chaetomium* and *Condenascus* were primarily involved in positive correlations with other taxa, whereas *Volutella* was largely associated with negative interactions. Other genera of note included *Tausonia, Fusarium, Pseudoacrodictys, Hyalopeziza, Linnemannia, Cladosporium*, and *Stachybotrys* (Figure 6b and Supplementary Tables S13, S14).

Further network analysis revealed that inoculant applications significantly influenced the topology of both bacterial and fungal communities (Figure 6c and Supplementary Tables S15, S16). In bacterial networks, graph density was consistently higher in all inoculant-treated groups (T1–T4: 0.084–0.112) than in the control (CK: 0.069). The density peaked in T2 (0.112) and then gradually decreased through T3 to T4. Similarly, the proportion of positive correlations in bacterial networks was elevated in the inoculant treatments (52.29%−57.14%) compared to CK (48.20%). Among the treatments, T2 showed the highest percentage (57.14%), followed by T3 (54.96%), T1 (53.14%), and T4 (52.29%). For fungal networks, inoculant treatments also resulted in higher graph density (T1–T4: 0.076–0.102) than CK (0.061), with the maximum density observed in T2 (0.102). The percentage of positive correlations was consistently greater in the inoculant groups (62.83%−69.53%) than in CK (53.15%), with the highest value in T2 (69.53%), followed by T1 (68.90%), T3 (66.19%), and T4 (62.83%).

To further elucidate the relationships between microbial communities and soil characteristics, Spearman correlation analysis was performed between the top 50 bacterial and fungal genera and soil physicochemical properties. Within bacterial communities, the majority of genera (76.32%) exhibited positive correlations with soil properties, while 23.68% showed negative correlations. Notably, several beneficial bacterial genera demonstrated significant positive correlations with key soil physicochemical indicators. Specifically, *Bacillus* and *Pseudomonas* showed strong positive correlations (ρ = 1, *p*_−adj_ < 0.001) with multiple soil properties including SOM, sucrase, AP, AK, and EC. *Flavobacterium* exhibited a significant positive correlation with urease (ρ = 1, *p*_−adj_ < 0.001), while *Nitrospira* was positively correlated with available nitrogen (AN) (ρ = 0.9, *p*_−adj_ < 0.05). Consistent with these correlations, the relative abundances of these taxa were markedly increased following microbial inoculant application (Figure 6d and Supplementary Table S17). In contrast, within the fungal community, only a smaller proportion (29.33%) of genera was positively correlated with soil properties, whereas the majority (70.67%) exhibited negative correlations. *Mortierella* showed significant positive correlations with multiple soil properties (SOM: ρ = 1, *p*_−adj_ < 0.001; sucrase: ρ = 1, *p*_−adj_ < 0.001). Conversely, several fungal genera demonstrated significant negative correlations: *Tausonia, Penicillium*, and related taxa were negatively correlated with SOM and multiple enzyme activities (ρ = −0.9 to −1, *p*_−adj_ < 0.05), while *Linnemannia* showed a strong negative correlation with pH (ρ = −1, *p*_−adj_ < 0.001) (Figure 6e and Supplementary Table S18). All significant correlations (*p*_−adj_ < 0.05) with their corresponding correlation coefficients (ρ) are fully reported in Supplementary Tables S17, S18.

## 4 Discussion

Soil microorganisms are essential components of agricultural ecosystems, and play a vital role in maintaining soil fertility and ecosystem function. Microbial inoculants application enhances the sustainability of agricultural systems by improving soil health and quality, promoting plant growth, enhancing stress resistance, and improving fruit quality. Furthermore, a growing body of evidence suggests that the introduction of specific microbial consortia can effectively reshape the composition of soil microbial communities, thereby contributing to more resilient and sustainable agricultural management systems. Based on the findings of this study, the application of microbial inoculants significantly altered the structure of the soil microbial community. Under moderate inoculation treatments (T1–T2), bacterial ASV richness (as indicated by Sobs, Chao1, and ACE indices) increased significantly, whereas excessive inoculation (T3–T4) led to a decline in bacterial abundance, likely due to resource competition or metabolic inhibition caused by over-colonization (Mawarda et al., 2020; Trabelsi and Mhamdi, 2013). Meanwhile, the Shannon diversity index and Pielou's evenness of the fungal community decreased across all treatments. This shift may be attributed to resource competition or microbial antagonism resulting from changes in bacterial community structure, indicating a higher sensitivity of fungi to inoculation disturbance.

In the grape rhizosphere bacterial community, several beneficial genera, including *Bacillus, Nitrospira, Pseudomonas, Flavobacterium, Bryobacter*, and *Planifilum*, were significantly enriched (Figure 5a, Supplementary Figure S3a, and Supplementary Table S10). Consistent with their established roles in the literature, genera such as *Bacillus* and *Pseudomonas* are known to contribute to phosphate solubilization, siderophore production, and induced systemic resistance (Bakki et al., 2024; Cochard et al., 2022), while *Nitrospira* is a well-documented nitrifier (Guo et al., 2017). Co-occurrence network analysis revealed widespread positive correlations between these key genera and soil physicochemical properties (Figures 6d, e), suggesting their potential involvement in nutrient cycling processes. Further network analysis of treatment-specific communities revealed that inoculant applications significantly influenced microbial interaction topology. In both bacterial and fungal networks, all inoculant-treated groups exhibited higher graph density and a greater proportion of positive correlations compared to the control, with the most pronounced enhancement consistently observed in the T2 treatment (Figure 6c). This indicates that a moderate inoculant dose fostered the most complex and cooperative microbial networks. Within the fungal community, pathogenic taxa in Ascomycota (such as *Fusarium* and *Penicillium*) were significantly suppressed, whereas beneficial genera in Mortierellomycota and Basidiomycota (e.g., *Mortierella* and *Solicoccozyma*) were markedly enriched (Figure 5b, Supplementary Figure S3b, and Supplementary Table S10). Network analysis further indicated that pathogenic fungi were mostly negatively correlated with beneficial fungi and soil physicochemical factors, whereas beneficial fungi showed positive correlations with soil nutrient indicators. As we all know, *Penicillium* are known postharvest pathogens of grape berries, causing blue mold rot and contaminating fruit with mycotoxins such as patulin, which poses significant risks to wine and juice quality (Chen et al., 2025; Ghuffar et al., 2021). Similarly, *Fusarium* species include soil-borne pathogens associated with grapevine decline and root rot, contributing to yield loss and reduced vineyard longevity (Akgül et al., 2024; Bustamante et al., 2024). Their reduction has a positive effect on soil health, as well as the health and growth of subsequent crops. In contrast, *Mortierella* suppresses pathogenic fungi such as *Fusarium* via antimicrobial metabolite secretion and niche competition (Wang et al., 2022), and enhances organic matter decomposition and nutrient release (Li et al., 2018). Basidiomycota also works on lignocellulose degradation and soil organic matter transformation (Valaskova et al., 2007), while its mycelial networks and extracellular polysaccharides help stabilize soil structure and maintain microecological balance (Lucas et al., 2014).

These shifts in microbial community structure demonstrated and interaction patterns that microbial inoculation promotes the enrichment of functional groups involved in nutrient cycling and pathogen suppression, while inhibiting taxa associated with organic carbon mineralization and pathogen synergism (Li et al., 2024a; Shen et al., 2021). Notably, the T2 treatment (90 L/ha) yielded the most favorable microecological outcomes: enhanced synergistic interactions among key bacterial taxa, suppression of pathogenic fungi, and significant enrichment of beneficial fungi, collectively contributing to improved rhizosphere micro-environment health and nutrient use efficiency (Arie, 2019; Ozimek and Hanaka, 2021).

Nutrient availability, including available phosphorus, rapidly available potassium, alkali-hydrolyzable nitrogen, and organic matter, constitutes a fundamental chemical indicator of soil fertility, which directly governs the soil's ability to sustain plant growth (Diallo et al., 2021). Soil enzyme activities, such as those of urease, alkaline phosphatase, and sucrase, are regarded as essential biological parameters for evaluating soil ecological function and health (Wang et al., 2023a). Their activity levels serve as direct proxies for the intensity of specific biochemical processes and the capacity for nutrient transformation and mobilization. Experimental results revealed that microbial inoculant applications (T1–T4) significantly enhanced both soil nutrient availability and enzymatic activities (Figure 1). This phenomenon closely linked to the reconfiguration of microbial community structure, and showed by the correlation analysis in Figure 6c. Notably, the T2 treatment demonstrated superior nutrient availability and soil enzyme activities compared to other treatments. This improvement coincided with the most pronounced enrichment of beneficial microorganisms and the strongest suppression of harmful microbes in the rhizospheric microbial community. These findings suggest that an insufficient inoculation dose (T1) may fail to maximize benefits, while excessive application (T3, T4) could disrupt microbial balance through mechanisms such as competitive exclusion or the accumulation of metabolic by-products (Trabelsi and Mhamdi, 2013). Therefore, an optimal inoculation dosage is crucial.

The results demonstrated that microbial inoculation significantly enhanced grape stress resistance by improving key physiological parameters in leaves (Figure 2). Specifically, the T2 treatment (90 L/ha) exhibited the most pronounced effects, with significant increases in antioxidant enzyme activities (POD, SOD, CAT) and proline content, coupled with a marked reduction in malondialdehyde (MDA) levels compared to other treatments. These findings indicate that the T2 treatment effectively mitigated membrane lipid peroxidation and improved osmotic regulation in grape plants. The observed physiological improvements may be linked to shifts in the rhizosphere microbial community structure. The T2 treatment promoted a more interconnected microbial network (Figure 6c), characterized by an increase in the relative abundance of beneficial taxa and a decrease in harmful ones (Figures 5a, b). These structural changes likely contributed to enhanced plant stress tolerance through several mechanisms: (1) improving soil nutrient availability (Figure 1), thereby facilitating the synthesis of antioxidants and osmoregulatory compounds; and (2) potentially modulating plant stress response pathways via systemic induction of resistance. These results are consistent with previous studies by He et al. and Singh et al., further supporting the proposed “soil microbial community–soil environment–plant physiology” cascade (He et al., 2024b; Singh et al., 2020). The synchronized improvements in soil quality, microbial community structure, and plant physiological indicators under the T2 treatment underscore the role of optimized microbial inoculation in enhancing grape stress resilience through rhizosphere microbiome remodeling.

Microbial inoculant application significantly improved both external and internal quality attributes of grape berries (Figure 3 and Table 1). After inoculation treatment, key external quality parameters, including fresh weight, dry weight, transverse diameter, and longitudinal diameter, showed consistent enhancements, and internal nutritional quality, such as soluble sugars, titratable acidity, soluble protein, and vitamin C content, was also markedly increased. Notably, the T2 treatment yielded superior quality grapes compared to the other treatments. This improvement can likely be attributed to the imporved microecological environment observed in T2, which was characterized by the most favorable soil properties and the healthiest leaf physiological condition. This synergistic advantage may positively regulate fruit development and quality formation by promoting plant nutrient absorption and metabolic efficiency. Figure 6 shows a significant correlation between fruit quality improvement and root zone environment improvement, indicating that rhizosphere ecology plays a key role in quality regulation. In particular, a functionally structured microbial community may facilitate nutrient mineralization and availability, stimulate phytohormone synthesis, and elicit systemic resistance against pathogens, which contribute to improved fruit yield and quality. These findings align with earlier studies reporting that microbial inoculants can positively modulate soil nutrient dynamics and plant performance, leading to higher productivity and better-quality harvests (Li et al., 2025; Shi et al., 2024).

Overall, the results from this study highlight significant correlations between soil microbial community structure, soil physicochemical properties, and plant physiological and fruit responses. These findings suggest the potential of microbial inoculants to enhance soil quality, plant stress resistance, and fruit quality through the restructuring of rhizosphere microbial communities. However, we explicitly acknowledge that these interpretations are constrained by the reliance on 16S/ITS amplicon sequencing data, which reveals taxonomic composition but does not directly demonstrate functional microbial processes. Consequently, the proposed mechanisms linking microbial taxa to ecosystem functions remain hypothetical and require validation through integrated functional approaches, such as metagenomics, metabolomics, and long-term field trials. Future research should therefore prioritize identifying the specific functional groups and metabolic pathways responsible for the observed benefits, and explore practical strategies for precisely manipulating the soil microbiome to enhance vineyard sustainability.

## 5 Conclusions

In conclusion, our study demonstrates that microbial inoculant application effectively reshapes the rhizosphere microbial community structure by enriching beneficial taxa and suppressing potential pathogens. These structural shifts were strongly associated with improved soil quality, enhanced plant stress resistance, and elevated fruit quality. These observed improvements followed a distinct dose-dependent pattern, with the 90 L/ha application rate conferring optimal performance across most measured parameters in grape production. We emphasiz, however, that the causal mechanisms linking these microbial community changes to plant phenotypes remain inferential and require validation through integrated functional approaches and require validation through integrated functional approaches. Future research should prioritize metagenomic and metabolomic analyses to directly characterize functional genes and metabolic pathways, combined with long-term field trials to verify the ecological consistency and agricultural relevance of these interactions under practical viticulture conditions. Such studies would substantially advance our understanding of dose-dependent plant-microbe-environment interactions and support the development of precision microbial management strategies for sustainable viticulture.

## Data Availability

The datasets presented in this study can be found in online repositories. The names of the repository/repositories and accession number(s) can be found in the article/Supplementary material.
