# Supplementary material for: Dose-optimized microbial inoculants reshape grape rhizosphere microbiota and enhance fruit quality
Source: Front Microbiol. 2025 Nov 5;16:1702884. doi: 10.3389/fmicb.2025.1702884 (PMC12626922; doi:10.3389/fmicb.2025.1702884)
Supplement: Supplementary file 1 [file Table_1.docx]

**Supplementary Material**

**Dose-Optimized Microbial Inoculants Reshape Grape Rhizosphere Microbiota and Enhance Fruit Quality**

Xiaojian Chang^1#^, Ji Chen^2#^, Kegang Zhao^1^, Tao Wang^1^, Yong Yang^1^， Xinyue Jia^2^, Bingbing Hu^2^, Fangxiang Li^1^, Yanmei Yu^1^, Yanhui He^2^*, Zhansheng Wu^2^*

1. Agricultural Technology Extension Center of Xi’an, Xi’an 710048, P.R. China.

2. Xi’an Key Laboratory of Textile Chemical Engineering Auxiliaries, School of Environmental and Chemical Engineering, Xi’an Polytechnic University, Xi’an 710048, P.R. China.

* Corresponding author: Zhansheng Wu, Tel: 86029 62779279, Fax: 86029 62779281, E-mail address: [wuzhans@xpu.edu.cn](mailto:wuzhans@xpu.edu.cn). Yanhui He, E-mail address: yhhe@xpu.edu.cn.

#: Co-first author

Table S1

Baseline physicochemical properties of cherry orchard soil.

| Parameter | Value | Unit |
| --- | --- | --- |
| pH | 6.39 | - |
| Electrical conductivity | 13.56 | μS/cm |
| Soil organic matter | 1.32 | % |
| Available P | 165.83 | mg/kg |
| Available K | 384.00 | mg/kg |
| Alkali-hydrolyzable N | 146.38 | mg/kg |

Table S2

Effects of different inoculant application rates on grape rhizosphere soil physicochemical properties.

|  | pH | EC (μs/cm) | SOM (%) | Ak(mg/kg) | AN (mg/kg) | AP (mg/kg) | ALP (mg/g/d) | Urease (mg/g/d) | Sucrase (mg/g/d) |
| --- | --- | --- | --- | --- | --- | --- | --- | --- | --- |
| CK | 6.22±0.21a | 13.58±0.24c | 1.43±0.03d | 471.67±8.33d | 122.46±3.26c | 263.82±4.59d | 2.45±0.13c | 2.45±0.13c | 62.95±1.60c |
| T1 | 6.27±0.12a | 15.26±0.31b | 2.33±0.06c | 522.67±13.01c | 132.21±3.53b | 299.98±5.36c | 2.83±0.06b | 2.83±0.06b | 74.01±2.04b |
| T2 | 6.26±0.16a | 17.50±0.59a | 3.22±0.20a | 667.00±14.93a | 179.33±2.79a | 422.46±9.18a | 3.28±0.16a | 3.28±0.16a | 98.13±3.91a |
| T3 | 6.40±0.15a | 17.09±0.31a | 3.02±0.06a | 609.33±12.01b | 182.86±2.51a | 412.25±9.82a | 2.81±0.11b | 2.81±0.11b | 78.90±3.95b |
| T4 | 6.31±0.24a | 16.48±0.45a | 2.75±0.07b | 523.00±8.19c | 184.39±3.47a | 354.71±7.87b | 2.91±0.08b | 2.91±0.08b | 74.60±3.14b |

Note: CK (no inoculant treatment), T1 (45 L/ha inoculant), T2 (90 L/ha inoculant), T3 (135 L/ha inoculant), T4 (180 L/ha inoculant). Different lowercase letters represent significant differences between different treatments in the same column. All statistical analyses were based on three biological replicates (n = 3) per treatment.

Table S3

One-way ANOVA results of soil physicochemical properties and enzyme activities in grape rhizosphere soil under different microbial inoculant application rates.

|  | | Sum of Squares (SS) | Degrees of Freedom (df) | Mean Square (MS) | F value | p-value |
| --- | --- | --- | --- | --- | --- | --- |
| pH | Between Groups | 0.063 | 4 | 0.016 | 0.800 | 0.552 |
|  | Within Groups | 0.197 | 10 | 0.020 |  |  |
|  | Total | 0.260 | 14 |  |  |  |
| EC | Between Groups | 30.681 | 4 | 7.670 | 85.642 | 0.000 |
|  | Within Groups | 0.896 | 10 | 0.090 |  |  |
|  | Total | 31.576 | 14 |  |  |  |
| SOM | Between Groups | 6.038 | 4 | 1.509 | 225.966 | 0.000 |
|  | Within Groups | 0.067 | 10 | 0.007 |  |  |
|  | Total | 6.105 | 14 |  |  |  |
| AK | Between Groups | 73320.933 | 4 | 18330.233 | 136.183 | 0.000 |
|  | Within Groups | 1346.000 | 10 | 134.600 |  |  |
|  | Total | 74666.933 | 14 |  |  |  |
| AN | Between Groups | 11016.036 | 4 | 2754.009 | 279.487 | 0.000 |
|  | Within Groups | 98.538 | 10 | 9.854 |  |  |
|  | Total | 11114.574 | 14 |  |  |  |
| AP | Between Groups | 57224.452 | 4 | 14306.113 | 244.621 | 0.000 |
|  | Within Groups | 584.827 | 10 | 58.483 |  |  |
|  | Total | 57809.280 | 14 |  |  |  |
| ALP | Between Groups | 1.044 | 4 | 0.261 | 20.729 | 0.000 |
|  | Within Groups | 0.126 | 10 | 0.013 |  |  |
|  | Total | 1.169 | 14 |  |  |  |
| Urease | Between Groups | 420.946 | 4 | 105.237 | 14.516 | 0.000 |
|  | Within Groups | 72.497 | 10 | 7.250 |  |  |
|  | Total | 493.444 | 14 |  |  |  |
| Sucrase | Between Groups | 9.885 | 4 | 2.471 | 189.039 | 0.000 |
|  | Within Groups | 0.131 | 10 | 0.013 |  |  |
|  | Total | 10.016 | 14 |  |  |  |

Table S4

Effects of different inoculant application rates on grape leaf physiological and biochemical indices.

|  | Proline(μg/g) | MDA（nmol/g） | Chlorophyll a（mg/g） | Chlorophyll b（mg/g） | Carotenoid（mg/g） | POD(U/(g.min)) | SOD(U/g.min) | CAT（U/g.min） |
| --- | --- | --- | --- | --- | --- | --- | --- | --- |
| CK | 93.34±0.92e | 44.56±0.71a | 2.09±0.05e | 1.31±0.11b | 2.52±0.10d | 867.55±39.38d | 477.41±14.66d | 91.37±10.15d |
| T1 | 128.07±3.89d | 35.04±3.43bc | 2.35±0.10d | 1.05±0.14c | 2.63±0.02cd | 1123.11±77.71c | 535.76±6.12c | 142.13±10.15d |
| T2 | 299.51±3.16a | 24.35±0.50d | 3.60±0.06c | 1.87±0.07a | 3.08±0.03a | 1459.11±94.38ab | 612.77±12.24a | 159.05±5.86c |
| T3 | 232.03±1.97b | 35.93±2.46bc | 3.57±0.03a | 1.72±0.09a | 2.79±0.06b | 1512.00±89.81a | 608.71±20.68a | 162.43±10.15a |
| T4 | 165.49±2.50c | 36.98±1.48b | 3.12±0.04b | 1.43±0.12b | 2.74±0.12bc | 1338.67±19.64b | 580.34±122.24b | 148.90±15.50b |

Note: CK (no inoculant treatment), T1 (45 L/ha inoculant), T2 (90 L/ha inoculant), T3 (135 L/ha inoculant), T4 (180 L/ha inoculant). Different lowercase letters represent significant differences between different treatments in the same column. All statistical analyses were based on three biological replicates (n = 3) per treatment.

Table S5

One-way ANOVA results of grape leaf physiological and biochemical indices under different microbial inoculant application rates.

|  | | Sum of Squares (SS) | Degrees of Freedom (df) | Mean Square (MS) | F value | p-value |
| --- | --- | --- | --- | --- | --- | --- |
| Proline | Between Groups | 82014.488 | 4 | 20503.622 | 2837.732 | 0.000 |
|  | Within Groups | 72.254 | 10 | 7.225 |  |  |
|  | Total | 82086.742 | 14 |  |  |  |
| MDA | Between Groups | 5.843 | 4 | 1.461 | 359.693 | 0.000 |
|  | Within Groups | 0.041 | 10 | 0.004 |  |  |
|  | Total | 5.883 | 14 |  |  |  |
| Chlorophyll a | Between Groups | 1.265 | 4 | 0.316 | 28.019 | 0.000 |
|  | Within Groups | 0.113 | 10 | 0.011 |  |  |
|  | Total | 1.378 | 14 |  |  |  |
| Chlorophyll b | Between Groups | 11.882 | 4 | 2.970 | 655.231 | 0.000 |
|  | Within Groups | 0.045 | 10 | 0.005 |  |  |
|  | Total | 11.927 | 14 |  |  |  |
| Carotenoid | Between Groups | 0.530 | 4 | 0.133 | 22.496 | 0.000 |
|  | Within Groups | 0.059 | 10 | 0.006 |  |  |
|  | Total | 0.589 | 14 |  |  |  |
| CAT | Between Groups | 9934.333 | 4 | 2483.583 | 21.271 | 0.000 |
|  | Within Groups | 1167.588 | 10 | 116.759 |  |  |
|  | Total | 11101.921 | 14 |  |  |  |
| POD | Between Groups | 846275.349 | 4 | 211568.837 | 42.399 | 0.000 |
|  | Within Groups | 49899.333 | 10 | 4989.933 |  |  |
|  | Total | 896174.682 | 14 |  |  |  |
| SOD | Between Groups | 38805.653 | 4 | 9701.413 | 48.736 | 0.000 |
|  | Within Groups | 1990.620 | 10 | 199.062 |  |  |
|  | Total | 40796.273 | 14 |  |  |  |

Table S6

Effects of different inoculant application rates on the apparent quality of grape fruits.

|  | Fresh weight（g） | Dry weight（g） | Fruit firmness（kg/cm） | Transverse Diameter(mm) | Longitudinal Diameter(mm) | Fruit Shape Index（mm/mm） |
| --- | --- | --- | --- | --- | --- | --- |
| CK | 9.50±1.17c | 1.80±0.23c | 0.49±0.04c | 24.12±1.17a | 28.04±1.52c | 1.16±0.05ab |
| T1 | 12.22±1.04b | 1.82±0.27c | 0.56±0.02b | 27.81±1.87b | 29.62±1.95c | 1.07±0.10cd |
| T2 | 15.69±2.72a | 2.33±0.08ab | 0.64±0.05a | 28.05±3.54b | 33.32±2.39a | 1.21±0.22a |
| T3 | 14.61±1.04a | 2.31±0.25ab | 0.58±0.06b | 28.43±1.29b | 31.24±1.77b | 1.10±0.07bcd |
| T4 | 14.25±1.24a | 1.97±0.13bc | 0.57±0.06b | 27.45±1.48b | 31.34±3.13b | 1.14±0.08abc |

Note: CK (no inoculant treatment), T1 (45 L/ha inoculant), T2 (90 L/ha inoculant), T3 (135 L/ha inoculant), T4 (180 L/ha inoculant). Different lowercase letters represent significant differences between different treatments in the same column. All statistical analyses were based on three biological replicates (n = 3) per treatment.

Table S7

**One-way ANOVA results of the quality of grape fruits under different microbial inoculant application rates.**

|  |  | Sum of Squares (SS) | Degrees of Freedom (df) | Mean Square (MS) | F value | p-value |
| --- | --- | --- | --- | --- | --- | --- |
| Fresh weight | Between Groups | 494.641 | 4 | 123.660 | 51.432 | 0.000 |
|  | Within Groups | 228.411 | 95 | 2.404 |  |  |
|  | Total | 723.052 | 99 |  |  |  |
| Dry weight | Between Groups | 5.378 | 4 | 1.345 | 49.780 | 0.000 |
|  | Within Groups | 2.566 | 95 | 0.027 |  |  |
|  | Total | 7.944 | 99 |  |  |  |
| Transverse Diameter | Between Groups | 341.741 | 4 | 85.435 | 19.800 | 0.000 |
|  | Within Groups | 409.911 | 95 | 4.315 |  |  |
|  | Total | 751.652 | 99 |  |  |  |
| Longitudinal Diameter | Between Groups | 3731.550 | 4 | 932.888 | 178.620 | 0.000 |
|  | Within Groups | 496.162 | 95 | 5.223 |  |  |
|  | Total | 4227.712 | 99 |  |  |  |
| Fruit firmness | Between Groups | 0.241 | 4 | 0.060 | 44.324 | 0.000 |
|  | Within Groups | 0.129 | 95 | 0.001 |  |  |
|  | Total | 0.371 | 99 |  |  |  |
| Titratable acidity | Between Groups | 0.015 | 4 | 0.004 | 31.874 | 0.000 |
|  | Within Groups | 0.001 | 10 | 0.000 |  |  |
|  | Total | 0.016 | 14 |  |  |  |
| Soluble sugar | Between Groups | 39.659 | 4 | 9.915 | 32.553 | 0.000 |
|  | Within Groups | 3.046 | 10 | 0.305 |  |  |
|  | Total | 42.705 | 14 |  |  |  |
| Sugar-acid ratio | Between Groups | 483.848 | 4 | 120.962 | 87.472 | 0.000 |
|  | Within Groups | 13.829 | 10 | 1.383 |  |  |
|  | Total | 497.677 | 14 |  |  |  |
| Soluble sugar | Between Groups | 65.930 | 4 | 16.482 | 27.491 | 0.000 |
|  | Within Groups | 5.996 | 10 | 0.600 |  |  |
|  | Total | 71.925 | 14 |  |  |  |
| Soluble protein | Between Groups | 2339.560 | 4 | 584.890 | 35.478 | 0.000 |
|  | Within Groups | 164.862 | 10 | 16.486 |  |  |
|  | Total | 2504.422 | 14 |  |  |  |
| Anthocyanins | Between Groups | 16.899 | 4 | 4.225 | 28.648 | 0.000 |
|  | Within Groups | 1.475 | 10 | 0.147 |  |  |
|  | Total | 18.374 | 14 |  |  |  |
| Vitamin C | Between Groups | 151.298 | 4 | 37.824 | 124.054 | 0.000 |
|  | Within Groups | 3.049 | 10 | 0.305 |  |  |
|  | Total | 154.347 | 14 |  |  |  |

Table S8

**One-way ANOVA results of** Bacterial and fungal alpha diversity indices **under different microbial inoculant application rates.**

|  |  |  | Sum of Squares (SS) | Degrees of Freedom (df) | Mean Square (MS) | F value | p-value |
| --- | --- | --- | --- | --- | --- | --- | --- |
| Bacteria | Sobs | Between Groups | 46650.267 | 4 | 11662.567 | 8.674 | 0.003 |
|  |  | Within Groups | 13444.667 | 10 | 1344.467 |  |  |
|  |  | Total | 60094.933 | 14 |  |  |  |
|  | ACE | Between Groups | 54250.400 | 4 | 13562.600 | 8.425 | 0.003 |
|  |  | Within Groups | 16098.000 | 10 | 1609.800 |  |  |
|  |  | Total | 70348.400 | 14 |  |  |  |
|  | Chao 1 | Between Groups | 49579.333 | 4 | 12394.833 | 8.829 | 0.003 |
|  |  | Within Groups | 14038.667 | 10 | 1403.867 |  |  |
|  |  | Total | 63618.000 | 14 |  |  |  |
|  | Shannon | Between Groups | 0.026 | 4 | 0.007 | 0.581 | 0.684 |
|  |  | Within Groups | 0.112 | 10 | 0.011 |  |  |
|  |  | Total | 0.138 | 14 |  |  |  |
|  | Simpson | Between Groups | 0.000 | 4 | 0.000 | 2.400 | 0.119 |
|  |  | Within Groups | 0.000 | 10 | 0.000 |  |  |
|  |  | Total | 0.000 | 14 |  |  |  |
|  | Pielou_e | Between Groups | 0.000 | 4 | 0.000 | 0.375 | 0.821 |
|  |  | Within Groups | 0.002 | 10 | 0.000 |  |  |
|  |  | Total | 0.002 | 14 |  |  |  |
| Fungi | Sobs | Between Groups | 14993.500 | 4 | 3748.375 | 3.416 | 0.058 |
|  |  | Within Groups | 9874.500 | 9 | 1097.167 |  |  |
|  |  | Total | 24868.000 | 13 |  |  |  |
|  | ACE | Between Groups | 15922.167 | 4 | 3980.542 | 3.394 | 0.059 |
|  |  | Within Groups | 10555.833 | 9 | 1172.870 |  |  |
|  |  | Total | 26478.000 | 13 |  |  |  |
|  | Chao 1 | Between Groups | 15255.024 | 4 | 3813.756 | 3.413 | 0.058 |
|  |  | Within Groups | 10055.833 | 9 | 1117.315 |  |  |
|  |  | Total | 25310.857 | 13 |  |  |  |
|  | Shannon | Between Groups | 0.631 | 4 | 0.158 | 4.882 | 0.023 |
|  |  | Within Groups | 0.291 | 9 | 0.032 |  |  |
|  |  | Total | 0.922 | 13 |  |  |  |
|  | Simpson | Between Groups | 0.001 | 4 | 0.000 | 10.742 | 0.002 |
|  |  | Within Groups | 0.000 | 9 | 0.000 |  |  |
|  |  | Total | 0.002 | 13 |  |  |  |
|  | Pielou_e | Between Groups | 0.009 | 4 | 0.002 | 4.442 | 0.030 |
|  |  | Within Groups | 0.004 | 9 | 0.000 |  |  |
|  |  | Total | 0.013 | 13 |  |  |  |

Table S9

**One-way ANOVA results of** the relative abundance of bacterial and fungal phyla **under different microbial inoculant application rates.**

|  | | Sum of Squares (SS) | Degrees of Freedom (df) | Mean Square (MS) | F value | p-value |
| --- | --- | --- | --- | --- | --- | --- |
| Proteobacteria | Between Groups | 115.994 | 4 | 28.999 | 11.270 | 0.001 |
|  | Within Groups | 25.732 | 10 | 2.573 |  |  |
|  | Total | 141.726 | 14 |  |  |  |
| Firmicutes | Between Groups | 126.378 | 4 | 31.595 | 44.277 | 0.000 |
|  | Within Groups | 7.136 | 10 | 0.714 |  |  |
|  | Total | 133.514 | 14 |  |  |  |
| Acidobacteriota | Between Groups | 183.750 | 4 | 45.937 | 38.027 | 0.000 |
|  | Within Groups | 12.080 | 10 | 1.208 |  |  |
|  | Total | 195.830 | 14 |  |  |  |
| Bacteroidota | Between Groups | 11.863 | 4 | 2.966 | 124.931 | 0.000 |
|  | Within Groups | 0.237 | 10 | 0.024 |  |  |
|  | Total | 12.101 | 14 |  |  |  |
| Actinobacteriot | Between Groups | 5.019 | 4 | 1.255 | 24.128 | 0.000 |
|  | Within Groups | 0.520 | 10 | 0.052 |  |  |
|  | Total | 5.539 | 14 |  |  |  |
| Chloroflexi | Between Groups | 10.389 | 4 | 2.597 | 74.849 | 0.000 |
|  | Within Groups | 0.347 | 10 | 0.035 |  |  |
|  | Total | 10.736 | 14 |  |  |  |
| Gemmatimonadota | Between Groups | 2.312 | 4 | 0.578 | 21.181 | 0.000 |
|  | Within Groups | 0.273 | 10 | 0.027 |  |  |
|  | Total | 2.585 | 14 |  |  |  |
| Verrucomicrobiota | Between Groups | 12.407 | 4 | 3.102 | 181.887 | 0.000 |
|  | Within Groups | 0.171 | 10 | 0.017 |  |  |
|  | Total | 12.578 | 14 |  |  |  |
| Myxococcota | Between Groups | 1.118 | 4 | 0.280 | 39.816 | 0.000 |
|  | Within Groups | 0.070 | 10 | 0.007 |  |  |
|  | Total | 1.188 | 14 |  |  |  |
| Nitrospirota | Between Groups | 1.130 | 4 | 0.282 | 49.197 | 0.000 |
|  | Within Groups | 0.057 | 10 | 0.006 |  |  |
|  | Total | 1.187 | 14 |  |  |  |
| Ascomycota | Between Groups | 780.945 | 4 | 195.236 | 35.912 | 0.000 |
|  | Within Groups | 54.364 | 10 | 5.436 |  |  |
|  | Total | 835.309 | 14 |  |  |  |
| Mortierellomycota | Between Groups | 522.959 | 4 | 130.740 | 47.231 | 0.000 |
|  | Within Groups | 27.681 | 10 | 2.768 |  |  |
|  | Total | 550.640 | 14 |  |  |  |
| Basidiomycota | Between Groups | 160.612 | 4 | 40.153 | 69.999 | 0.000 |
|  | Within Groups | 5.736 | 10 | 0.574 |  |  |
|  | Total | 166.349 | 14 |  |  |  |

Table S10

Kruskal-Wallis rank-sum test statistics for the top 50 most abundant bacterial and fungal genera across treatments.

| Bacteria |  |  |  | Fungi |  |  |  |
| --- | --- | --- | --- | --- | --- | --- | --- |
| Species name | Statistic | P_value | P_adjust | Species name | Statistic | P_value | P_adjust |
| Microvirga | 7.513 | 0.057 | 0.172 | Solicoccozyma | 11.033 | 0.026 | 0.036 |
| Lysobacter | 7.230 | 0.065 | 0.182 | Exophiala | 6.733 | 0.151 | 0.426 |
| Skermanella | 7.821 | 0.050 | 0.164 | Neocosmospora | 5.151 | 0.272 | 0.511 |
| Dongia | 7.615 | 0.055 | 0.170 | Mycoarthris | 9.367 | 0.053 | 0.310 |
| Sphingomonas | 9.974 | 0.019 | 0.039 | Tausonia | 11.133 | 0.025 | 0.036 |
| Acidibacter | 6.613 | 0.085 | 0.199 | Mortierella | 10.300 | 0.036 | 0.045 |
| Terrimonas | 6.692 | 0.082 | 0.199 | Fusarium | 10.513 | 0.033 | 0.045 |
| Bryobacter | 9.495 | 0.023 | 0.039 | Penicillium | 13.057 | 0.011 | 0.024 |
| Bacillus | 9.667 | 0.022 | 0.039 | Hormiactis | 11.433 | 0.022 | 0.036 |
| mle1-7 | 9.585 | 0.022 | 0.141 | Pyxidiophorales_gen_Incertae_sedis | 9.079 | 0.059 | 0.310 |
| Candidatus_Udaeobacter | 8.744 | 0.053 | 0.151 | Rozellomycota_gen_Incertae_sedis | 7.633 | 0.106 | 0.366 |
| Steroidobacter | 8.744 | 0.053 | 0.151 | Enterocarpus | 9.801 | 0.054 | 0.310 |
| Chryseolinea | 8.949 | 0.030 | 0.150 | Thelebolaceae_gen_Incertae_sedis | 9.501 | 0.060 | 0.310 |
| Hyphomicrobium | 9.667 | 0.072 | 0.141 | Cheilymenia | 6.813 | 0.146 | 0.426 |
| SWB02 | 9.392 | 0.065 | 0.141 | GS08_gen_Incertae_sedis | 7.403 | 0.116 | 0.374 |
| Novosphingobium | 8.231 | 0.055 | 0.151 | Fusicolla | 8.900 | 0.064 | 0.310 |
| Planifilum | 9.698 | 0.021 | 0.039 | Linnemannia | 11.567 | 0.021 | 0.036 |
| Gaiella | 8.465 | 0.037 | 0.151 | GS10_gen_Incertae_sedis | 6.735 | 0.151 | 0.426 |
| Altererythrobacter | 7.359 | 0.075 | 0.141 | Marquandomyces | 7.333 | 0.119 | 0.378 |
| Flavobacterium | 9.974 | 0.019 | 0.039 | Coprinellus | 3.977 | 0.409 | 0.513 |
| Hydrogenispora | 6.436 | 0.088 | 0.151 | Acrostalagmus | 2.367 | 0.669 | 0.683 |
| Tumebacillus | 9.513 | 0.023 | 0.141 | Sagenomella | 7.190 | 0.126 | 0.398 |
| Amaricoccus | 6.619 | 0.092 | 0.141 | Botryotrichum | 4.431 | 0.351 | 0.511 |
| TM7 | 7.872 | 0.069 | 0.164 | Pseudaleuria | 11.133 | 0.025 | 0.036 |
| Lysinibacillus | 7.430 | 0.064 | 0.141 | Stephanonectria | 9.436 | 0.054 | 0.310 |
| Solitalea | 7.566 | 0.056 | 0.172 | Aspergillus | 3.630 | 0.458 | 0.560 |
| Massilia | 8.452 | 0.038 | 0.151 | Ascobolus | 4.616 | 0.329 | 0.511 |
| Nordella | 6.154 | 0.097 | 0.143 | Trichosporon | 4.838 | 0.304 | 0.511 |
| Adhaeribacter | 8.077 | 0.054 | 0.155 | Holtermanniella | 6.932 | 0.140 | 0.426 |
| Romboutsia | 9.546 | 0.023 | 0.141 | Alfoldia | 6.356 | 0.174 | 0.466 |
| Aquicella | 8.273 | 0.061 | 0.151 | Clonostachys | 6.286 | 0.179 | 0.466 |
| Ensifer | 7.974 | 0.089 | 0.141 | Ochroconis | 10.820 | 0.029 | 0.310 |
| Agromyces | 7.848 | 0.049 | 0.164 | Acaulium | 6.279 | 0.179 | 0.466 |
| Thermoactinomyces | 7.462 | 0.054 | 0.141 | Lasiosphaeriaceae_gen_Incertae_sedis | 12.700 | 0.013 | 0.310 |
| Haliangium | 6.846 | 0.077 | 0.199 | Hogelandia | 10.392 | 0.034 | 0.310 |
| Sporosarcina | 7.631 | 0.106 | 0.366 | Cladosporium | 5.476 | 0.242 | 0.511 |
| Clostridium_sensu_stricto_1 | 7.009 | 0.088 | 0.141 | Cercophora | 7.680 | 0.104 | 0.366 |
| Turicibacter | 7.230 | 0.065 | 0.182 | Lophiotrema | 11.233 | 0.024 | 0.310 |
| Nitrosospira | 6.131 | 0.190 | 0.466 | Petriella | 9.079 | 0.059 | 0.310 |
| Pedomicrobium | 9.462 | 0.024 | 0.141 | Hyalopeziza | 5.004 | 0.287 | 0.511 |
| Edaphobaculum | 8.839 | 0.032 | 0.151 | Dactylonectria | 11.573 | 0.021 | 0.310 |
| Ureibacillus | 10.200 | 0.017 | 0.141 | Microascales_gen_Incertae_sedis | 11.035 | 0.026 | 0.310 |
| Neochlamydia | 7.333 | 0.119 | 0.378 | Bipolaris | 3.238 | 0.519 | 0.560 |
| Streptomyces | 4.900 | 0.298 | 0.511 | Gibellulopsis | 7.700 | 0.103 | 0.366 |
| Allorhizobium-Neorhizobium-Pararhizobium-Rhizobium | 8.504 | 0.075 | 0.316 | Pseudoacrodictys | 11.531 | 0.021 | 0.310 |
| Pseudomonas | 10.421 | 0.015 | 0.031 | Cephaliophora | 10.657 | 0.013 | 0.045 |
| Nitrospira | 9.701 | 0.021 | 0.039 | Thelebolus | 8.571 | 0.073 | 0.310 |
| Subgroup__10 | 5.100 | 0.277 | 0.511 | Trichothecium | 3.231 | 0.520 | 0.560 |
| Paenibacillus | 9.267 | 0.055 | 0.310 | Holtermannia | 5.593 | 0.232 | 0.511 |
| RB41 | 8.231 | 0.041 | 0.048 | Hohenbuehelia | 7.990 | 0.092 | 0.346 |

Table S11. Bacterial co-occurrence network node attributes.

Table S12. Bacterial co-occurrence network centrality metrics.

Table S13. Fungal co-occurrence network node attributes.

Table S14. Fungal co-occurrence network centrality metrics.

Note: Tables S11 to S14 are detailed in the supplementary material Excel file.

Table S15

Topological properties of bacterial co-occurrence networks across treatments.

|  | CK | T1 | T2 | T3 | T4 |
| --- | --- | --- | --- | --- | --- |
| Number of nodes | 90 | 95 | 85 | 84 | 89 |
| Number of edges | 278 | 382 | 399 | 353 | 327 |
| Number of positive edge (%) | 48.20 | 53.14 | 57.14 | 54.96 | 52.29 |
| Number of negitive edge (%) | 51.80 | 46.86 | 42.86 | 45.04 | 47.71 |
| Average degrees | 6.178 | 8.042 | 8.740 | 8.405 | 7.348 |
| Network diameter | 11 | 10 | 10 | 10 | 11 |
| Network density | 0.069 | 0.086 | 0.112 | 0.101 | 0.084 |
| Modularity | 0.699 | 0.644 | 0.627 | 0.645 | 0.675 |
| Average clustering coefficient | 0.577 | 0.577 | 0.622 | 0.604 | 0.613 |
| Characteristic path length | 4.613 | 4.023 | 3.807 | 3.929 | 4.623 |

Note: CK (no inoculant treatment), T1 (45 L/ha inoculant), T2 (90 L/ha inoculant), T3 (135 L/ha inoculant), T4 (180 L/ha inoculant).

Table S16

Topological properties of fungal co-occurrence networks across treatments.

|  | CK | T1 | T2 | T3 | T4 |
| --- | --- | --- | --- | --- | --- |
| Number of nodes | 90 | 87 | 82 | 89 | 88 |
| Number of edges | 244 | 283 | 338 | 352 | 304 |
| Number of positive edge (%) | 53.15 | 68.90 | 69.53 | 66.19 | 62.83 |
| Number of negitive edge (%) | 46.85 | 31.10 | 30.47 | 33.81 | 37.17 |
| Average degrees | 5.422 | 6.506 | 8.244 | 8.063 | 6.909 |
| Network diameter | 8 | 9 | 7 | 8 | 8 |
| Network density | 0.061 | 0.076 | 0.102 | 0.090 | 0.079 |
| Modularity | 0.633 | 0.582 | 0.544 | 0.572 | 0.61 |
| Average clustering coefficient | 0.404 | 0.415 | 0.531 | 0.531 | 0.529 |
| Characteristic path length | 3.806 | 3.832 | 3.403 | 3.483 | 3.596 |

Note: CK (no inoculant treatment), T1 (45 L/ha inoculant), T2 (90 L/ha inoculant), T3 (135 L/ha inoculant), T4 (180 L/ha inoculant).

Table S17. Spearman correlation analysis between dominant bacterial genera and soil physicochemical properties.

Table S18. Spearman correlation analysis between dominant fungal genera and soil physicochemical properties.

Note: Tables 2 to 5 are detailed in the supplementary material Excel file.


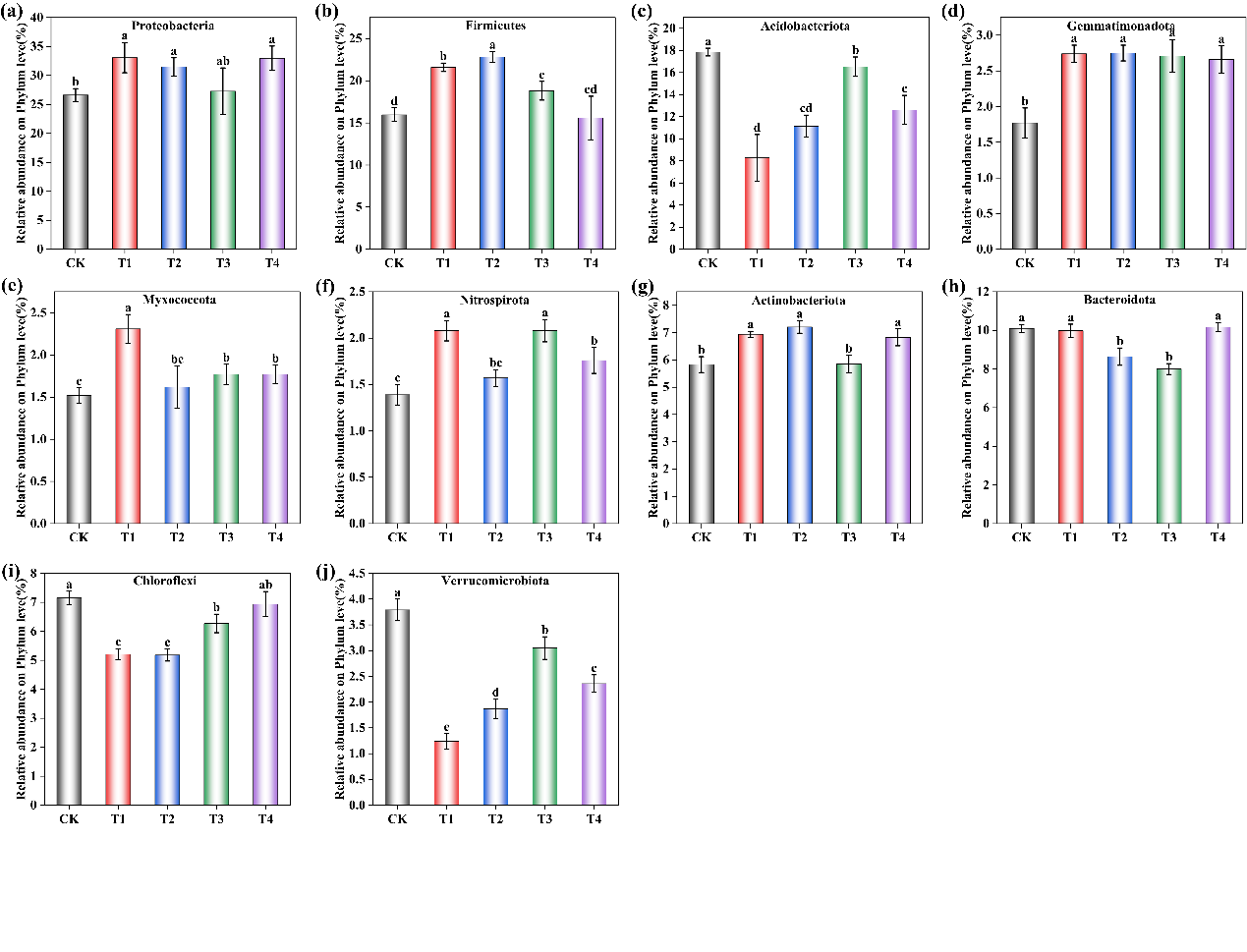


Fig. S1. Changes in the relative abundance of bacterial phyla under different fertilization regimes. (a) *Proteobacteria*;(b) *Firmicutes*;(c) *Actinobacteriota*; (d) *Gemmatimonadota*;(e) *Myxococcota*;(f) *Nitrospirota*;(g) *Acidobacteriota*;(h) *Bacteroidota*; (i) *Chloroflexi*; (j) *Verrucomicrobiota*. CK (no inoculant treatment), T1 (45 L/ha inoculant), T2 (90 L/ha inoculant), T3 (135 L/ha inoculant), T4 (180 L/ha inoculant). Bars and lines represent mean values of three replicates ± SE (standard error). A different letter at the head of a column indicates a significant difference (p < 0.05) from other treatments.


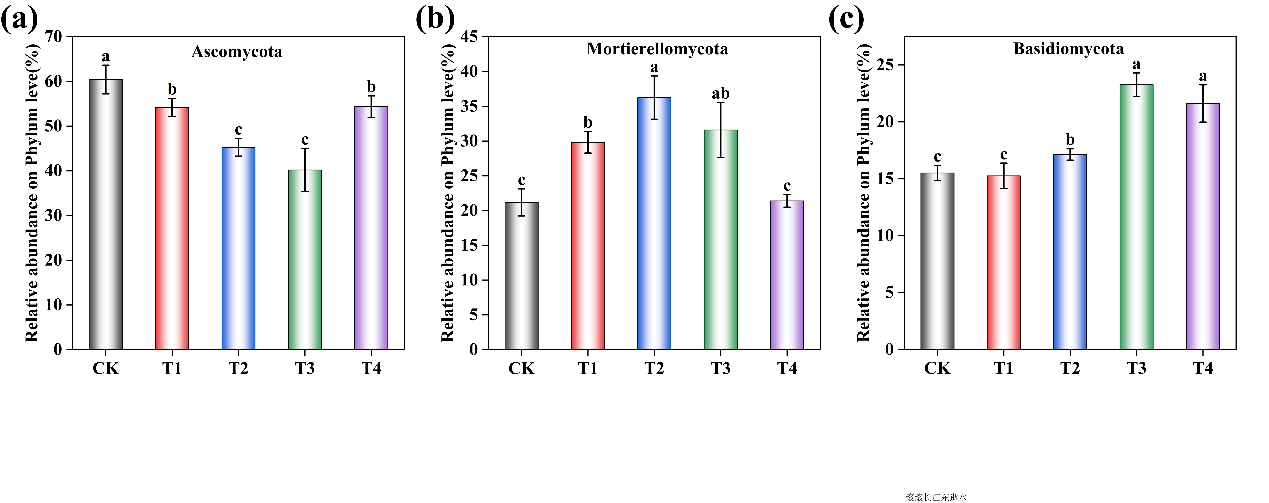


Fig. S2. Changes in the relative abundance of fungal phyla under different fertilization regimes. (a) Ascomycota; (b) Mortierellomycota; (c) Basidiomycota. CK (no inoculant treatment), T1 (45 L/ha inoculant), T2 (90 L/ha inoculant), T3 (135 L/ha inoculant), T4 (180 L/ha inoculant). Bars and lines represent mean values of three replicates ± SE (standard error). A different letter at the head of a column indicates a significant difference (p < 0.05) from other treatments.


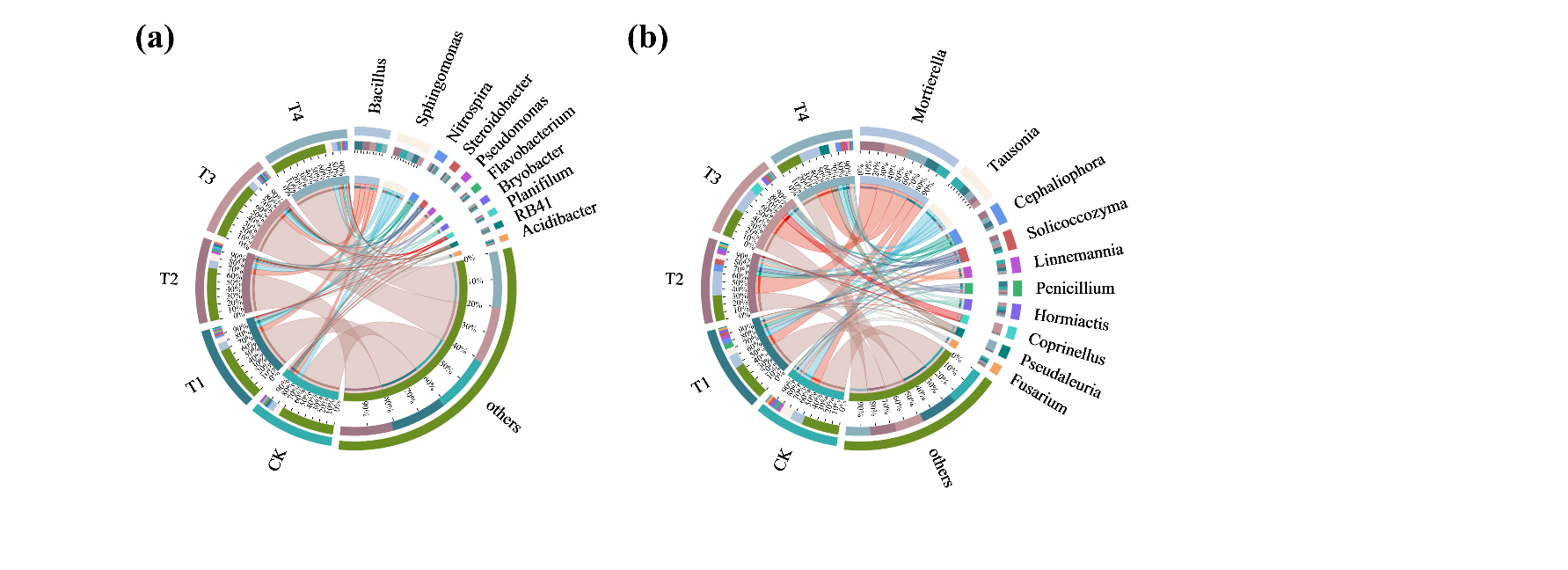
Fig. S3. Circos diagrams illustrating genus-level taxonomic distributions under different fertilization regimes. (a) Bacterial communities; (b) Fungal communities. CK (no inoculant treatment), T1 (45 L/ha inoculant), T2 (90 L/ha inoculant), T3 (135 L/ha inoculant), T4 (180 L/ha inoculant).
